# Willingness to take COVID-19 vaccination in low-income countries: Evidence from Ethiopia

**DOI:** 10.1371/journal.pone.0264633

**Published:** 2022-03-03

**Authors:** Christoph Strupat, Zemzem Shigute, Arjun S. Bedi, Matthias Rieger

**Affiliations:** 1 German Development Institute/Deutsches Institut für Entwicklungspolitik (DIE), Bonn, Germany; 2 Institute of Development and Policy Research, Addis Ababa University, Addis Ababa, Ethiopia; 3 International Institute of Social Studies, Erasmus University Rotterdam, Rotterdam, The Netherlands; KEMRI Wellcome Trust Research Programme, KENYA

## Abstract

**Background:**

In low-income countries, vaccination campaigns are lagging, and evidence on vaccine acceptance, a crucial public health planning input, remains scant. This is the first study that reports willingness to take COVID-19 vaccines and its socio-demographic correlates in Ethiopia, Africa’s second most populous country.

**Methods:**

The analysis is based on a nationally representative survey data of 2,317 households conducted in the informal economy in November 2020. It employs two logistic regression models where the two outcome variables are (i) a household head’s willingness to take a COVID-19 vaccine or not, and (ii) if yes if they would also hypothetically pay (an unspecified amount) for it or not. Predictors include age, gender, education, marital status, income category, health insurance coverage, sickness due to COVID-19, chronic illness, trust in government, prior participation in voluntary activities, urban residence.

**Results:**

Willingness to take the vaccine was high (88%) and significantly associated with COVID-19 cases in the family, trust in government and pro-social behavior. All other predictors such as gender, education, income, health insurance, chronic illness, urban residence did not significantly predict vaccine willingness at the 5% level. Among those willing to take the vaccine, 33% also answered that they would hypothetically pay (an unspecified amount) for it, an answer that is significantly associated with trust in government, health insurance coverage and income.

**Conclusion:**

The results highlight both opportunities and challenges. There is little evidence of vaccine hesitancy in Ethiopia among household heads operating in the informal economy. The role played by trust in government and pro-social behavior in motivating this outcome suggests that policy makers need to consider these factors in the planning of COVID-19 vaccine campaigns in order to foster vaccine uptake. At the same time, as the willingness to hypothetically pay for a COVID-19 vaccine seems to be small, fairly-priced vaccines along with financial support are also needed to ensure further uptake of COVID-19 vaccines.

## Introduction

A growing body of research examines the willingness to take COVID-19 vaccinations in high- and middle-income countries [1–7]. The evidence base from low-income countries (LICs) remains small, with a handful of studies from Sub Saharan Africa (SSA) [8–15]. Economic limitations and ill-functioning health systems hinder the ability of LICs to freely offer COVID-19 vaccines. In the absence of substantial international support, large shares of the population may have to rely on out-of-pocket-expenditures (OOP) to access the vaccine [16]. A few studies have also explored the willingness to pay for COVID-19 vaccines in LICs with almost no evidence from SSA [1, 8, 16–20]. Moreover, most of the reviewed studies [1–7, 9] have relied on phone and online surveys. Limited access to internet and poor phone networks in the case of LICs and the high likelihood that individuals residing outside the country of research are included in online social media based surveys potentially limit the validity of findings from such surveys [21]. Furthermore, even evidence from face-to-face surveys is either often restricted to respondents either residing in urban areas or engaged in the formal sector [11, 22, 23] or target a specific segment of the population [24, 25]. Our study contributes to closing this potential evidence gap by investigating the willingness to take COVID-19 vaccines based on a nationally representative in-person survey of the informal economy covering 2,317 households in Ethiopia. As 80% of all economic activities are part of the informal economy in Ethiopia [26], the survey covers the majority of the Ethiopian population. The informal economy is defined as all economic activities by workers and economic units that are—in law or in practice—not covered or insufficiently covered by formal arrangements [27].

With the aim of successful control of the pandemic [1, 28], this study informs COVID-19 vaccination campaigns in Ethiopia, and potentially other LICs with large informal economies, on the willingness to take vaccines and identifies its socio-demographic correlates.

As of December 1, 2021, Ethiopia had reported 371,672 confirmed cases of COVID-19 and 6,771 deaths with a case fatality rate of 1.82%. This amounts to 5.97% of all confirmed cases in WHO’s Africa region, placing the country second after South Africa in terms of the number of cases. Notwithstanding the encouraging recovery rate of about 93.9%, in terms of COVID-19 related deaths, the country ranks second in the WHO Africa region after South Africa [29, 30]. On March 13, 2021, the country launched a nationwide roll-out of the vaccine after receiving 2.2 million of the 7.62 million doses of SII AstraZeneca (COVISHIELD) secured by the government through COVAX. In addition, the country has received 1.34 million doses of AstraZeneca (AZD1222) directly donated by France (391,200) and UK (998,400), 1.66 million doses J&J/Janssen and 1.55 doses of Pfizer donated by USA, 1.8 million doses of Sinopharm donated by China and 108,000 doses of the 3 million doses of J&J procured by the Ethiopian government through the African Vaccine Acquisition Trust (AVAT) facility. Altogether the country has received and administered more than 10 million doses of different types of vaccines against COVID-19. Highlighting the limitations of a purely public-sector free vaccination campaign, the country anticipates covering 20% of its population by the end of 2021 [31].

## Methods

### Study and sample design

In this cross-sectional study, we collected information on the potential acceptance of a COVID-19 vaccine from 2,336 randomly selected households working in the informal economy. The objective of our survey was to obtain a better understanding of the social situation of households in the informal economy during the COVID-19 pandemic in Ethiopia, including attitudes on COVID-19 vaccines.

The sample is a representative cross-section of all members in the informal economy aged 15 or above. The informal economy is defined as all economic activities by workers and economic units that are—in law or in practice—not covered or insufficiently covered by formal arrangements [27]. The data was collected through in-person interviews in October and November 2020, after lockdown measures were eased in Ethiopia.

The sample universe associated with our survey includes all individuals in Ethiopia that are aged above 15 and that operate in the informal economy on the day of the survey. We exclude households that are operating in the formal economy. To obtain a nationally representative cross-section of this target population, we use the most recent national census data from the Central Statistics Agency (CSA) and the latest Labor Force Survey by the ILO as sampling frame. The sample size was determined so that the average margin of sampling error would not exceed |2%| at a 95% confidence level. This is the case with a randomly selected sample of 2,336 individuals. We used a clustered, stratified, multi-stage, probability sample design. The objective of our sample design was to give every individual aged 15 or above that operates in the informal economy an equal chance of being chosen for inclusion in the sample. This ensures that the survey provides a representative estimate of the views of the target population. We reached this objective by (a) strictly applying random selection methods at every stage of sampling and by (b) applying sampling with probability proportionate to adult population size. The sampling process was based on stratification of the country into regions. Regions were further classified into zones and these were further divided into woredas and kebeles. Woredas are districts and kebeles are sub-districts in the Ethiopian context. Primary sampling units (PSUs)—sometimes referred to as enumeration areas—are the smallest geographical unit/cluster for which reliable population data were obtainable. The primary sampling units were selected from each stratum based on shares of the national population, and further allocated based on the urban/rural divide. In total 292 enumeration areas were selected and a total of 8 households were surveyed per PSU (for details on the sampling process see S1 File).

### Questionnaire

Our questionnaire follows partly the Afrobarometer questionnaire for Ethiopia [32]. Questions on the COVID-19 pandemic were added, including a question on the willingness to take the COVID-19 vaccine. We used English as primary survey questionnaire language and translated all questions into the most widely spoken local languages in Ethiopia. All respondents were offered a choice of language in which they preferred to be asked questions. The questionnaire was administered to gather information from respondents after obtaining their oral informed consent about the survey. The oral consent was stored as an audio file. No personally identifiable information was collected nor stored.

### Study variables

Closely following previous work [1], the **independent variables** that we use for our analysis are the demographic and socioeconomic characteristics of the household head that included: age, gender, education status, monthly income, health insurance coverage, sickness due to COVID-19, chronic illness, trust in government, prior participation in voluntary activities and residence of the household.

The **dependent variables** were responses from the questionnaire about the willingness to take COVID-19 vaccines. Household heads were asked the question: *“If a vaccine for COVID-19 gets introduced*, *would you like to get it”*, and were offered three options–(1) no (2) yes, only for free and (3) yes, even if I have to pay. We analyze this question via two willingness to get the vaccine variables and in two steps: the first binary variable (labelled *Willingness to take COVID-19 vaccine*) takes on a value of 0 if the person answered (1 no) and 1 if answered (2 yes, only for free) or (3 yes, even if I have to pay). The second binary variable is an “augmented” willingness to take the vaccine indicator (labelled *Willingness to take COVID-19 vaccine even if having to pay for it [unspecified monetary amount and hypothetical]*) and takes on a value of 1 if the person answered (3 yes, even if I have to pay) and 0 if the person answered (2 yes, only for free). Respondents that were not willing to take the vaccine in the first place (1 no) are not included in this second part of the analysis. Also note that it was mechanically impossible to answer to be willing to pay for the COVID-19 vaccine but unwilling to take it.

Our simple question has several limitations: First, we did not include the answer option “undecided” which would, for instance, have indicated the share of respondents who potentially would be willing to take the vaccine after promotional activities. Second, the second variable should not be interpreted as willingness to pay (WTP). For a proper measurement of WTP, we would have had to present respondents with different monetary amounts and scenarios of vaccine availability. At the time of the survey, the vaccine was not widely available.

### Statistical analysis

To analyze the predictors of willingness to take COVID-19 vaccines, we employ a logistic regression model:

$$p(Y_{i}=1)=f\left({\beta X}_{i}\right),$$

where the probability (*p*(*Y*_*i*_ = 1)) that household head *i* is willing to take a COVID-19 vaccine is treated as a function of various characteristics (*X*_*i*_). For our analysis, we use the two aforementioned binary willingness to take the vaccine variables: (i) *Willingness to take COVID-19 vaccine* and (ii) *Willingness to take COVID-19 vaccine even if having to pay for it [unspecified monetary amount and hypothetical]*.

**Fig 1:**
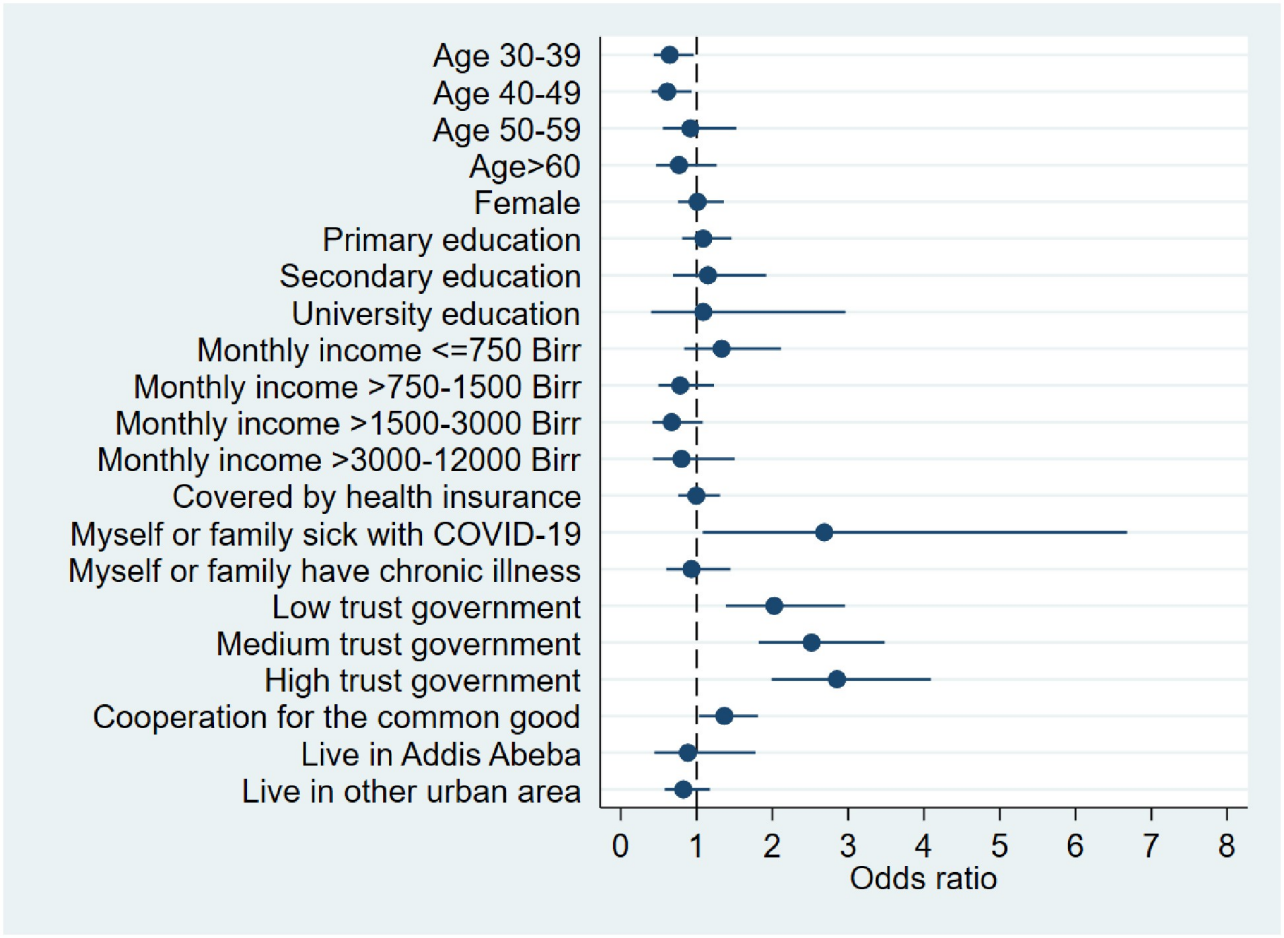
Willingness to take COVID-19 vaccine*. Fig 1 displays results from a logistic regression model of the willingness to take a COVID-19 vaccine. It depicts odds ratios (filled circles) and 95% confidence intervals for each of the explanatory variables (estimates are in S1 Table). The odds ratio with confidence interval for the highest income category [Monthly income >12000 Birr] is not displayed due to a large confidence interval. Estimates are based on 2,317 observations. * Based on the question: *“If a vaccine for COVID-19 gets introduced*, *would you like to get it”* and three exclusive answer options–(1) no (2) yes, only for free and (3) yes, even if I have to pay. Binary variable takes on a value of 0 if person answered (1 no) and 1 if answered (2 yes, only for free) or (3 yes, even if I have to pay).

**Fig 2:**
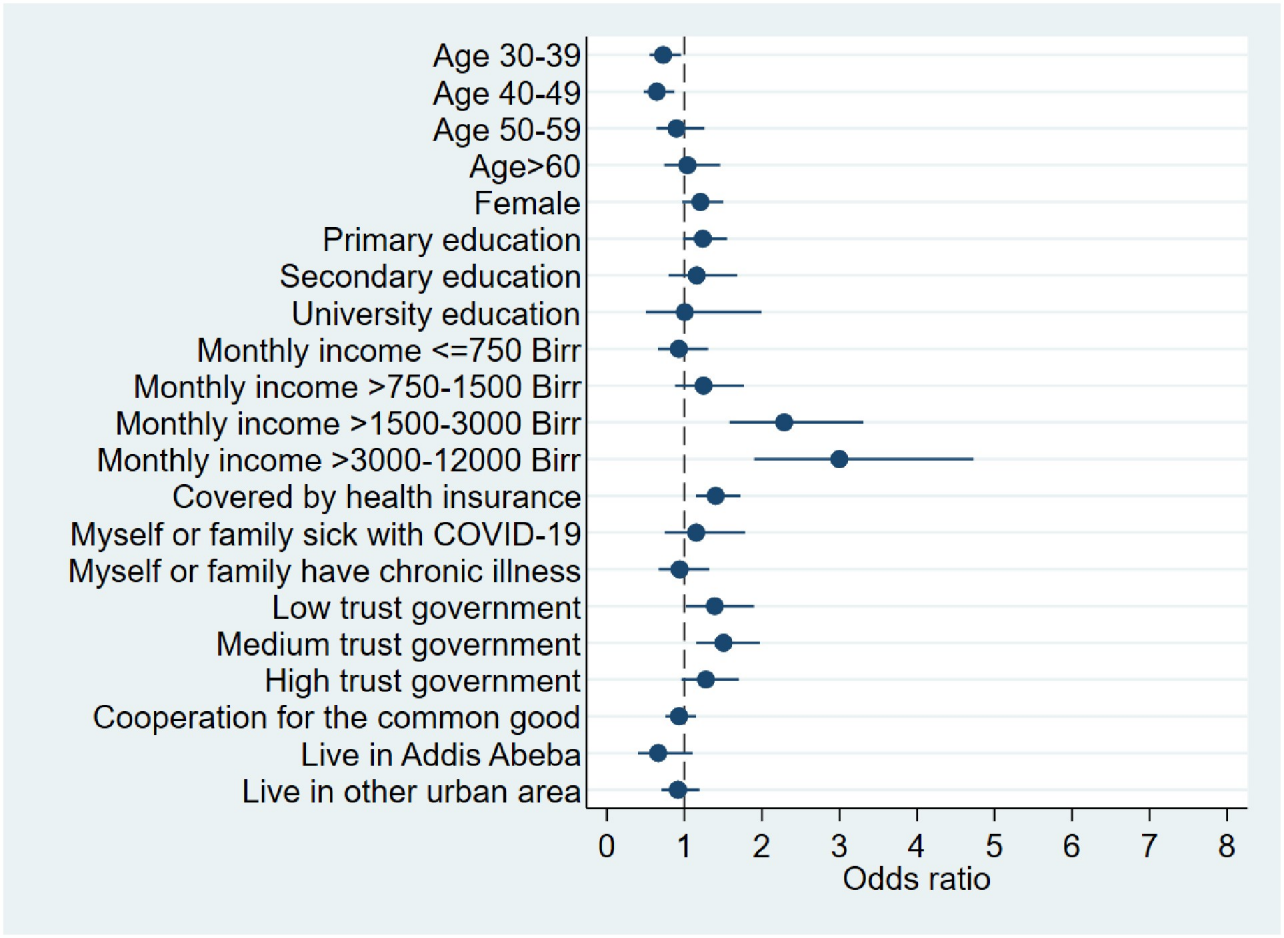
Willingness to take COVID-19 vaccine even if having to pay for it [unspecified monetary amount and hypothetical]*. Fig 2 displays results from a logistic regression model of the willingness to pay for a COVID-19 vaccine. It depicts odds ratios (filled circles and 95% confidence intervals for each of the explanatory variables (estimates are in S2 Table). The odds ratio with confidence interval for the highest income category [Monthly income >12000 Birr] is not displayed due to a large confidence interval. Estimates are based on 2,036 observations.- * Based on the question: *“If a vaccine for COVID-19 gets introduced*, *would you like to get it”* and three exclusive answer options–(1) no (2) yes, only for free and (3) yes, even if I have to pay. Binary variable takes on a value of 1 if the person answered (3 yes, even if I have to pay) and 0 if the person answered (2 yes, only for free). Respondents that were not willing to take the vaccine in the first place (1, no) are not included here. The variable should not be interpreted as a willingness to pay variable as question is hypothetical and no monetary amount was specified.

Both variables are regressed on a matrix of predictors (*X*_*i*_*)* hypothesized to be associated with the willingness to take and pay for a COVID-19 vaccine. Following previous work [1], the predictors include: age, gender, education, income category, health insurance coverage, sickness due to COVID-19, chronic illness, trust in government, prior participation in voluntary activities, urban residence (see detailed list of variables in S1 and S2 Tables). We report odds ratios along with standard errors, Z-statistics, p-values as well as 95% confidence intervals (in Figs 1 and 2) based on two-sided tests. The model was estimated using STATA 15.

Given the set of predictors, our results are based on 2,317 of 2,336 household heads on whom we have complete information on all predictors. This includes 4 households that refused to be part of the survey, the non-response rate is about 1%. For our first dependent variable the pseudo R square shows a goodness of fit of our model of 0.0482. If we focus on our second dependent variable the pseudo R square shows a goodness of fit of 0.0437. Furthermore, we checked for multicollinearity of the predictor variables and did not detect that the variance of the regression coefficients are inflated due to multicollinearity in the model (results available on request).

## Results

### Descriptive results

Descriptive statistics are in Table 1. Respondents were distributed across age groups as follows: 15–29 (22%), 30–39 (28%), 40–49 (23%), 50–59 (14%) and >60 years (13%). The majority of respondents were male (68%) and most had no schooling (38%) or only primary education (48%). About 12% and 35% of respondents reported no or less than 750 Birr (USD 19) monthly income, respectively. They overwhelmingly resided in rural areas (77%) as opposed to the capital (4%) or other urban areas (18%). An overwhelming majority of household heads (88%) were willing to take the vaccine. Positive answers were evenly distributed across rural (88%) and urban (87%) areas. Conditional on their willingness to take the vaccine, 33% responded that they were willing to take it and hypothetically pay (an unspecified amount) for it in the overall sample with 35% in rural and 32% in urban areas (see Table 2 for descriptive statistics and a breakdown by all covariates, respectively).

**Table 1:** Descriptive statistics.

Variable	Mean	Std. Dev.	Min	Max	Observation
**Outcome variables** *					
Willingness to take COVID-19 vaccine**	0.879	0.327	0	1	2,317
Rural	0.881	0.323	0	1	1,796
Urban	0.869	0.337	0	1	521
Willingness to take COVID-19 vaccine even if having to pay for it [unspecified monetary amount and hypothetical]***	0.328	0.469	0	1	2,036
Rural	0.322	0.467	0	1	1,583
Urban	0.347	0.476	0	1	453
**Covariates**					
Age 15–29	0.218	0.413	0	1	2,317
Age 30–39	0.281	0.450	0	1	2,317
Age 40–49	0.230	0.421	0	1	2,317
Age 50–59	0.136	0.343	0	1	2,317
Age >60	0.133	0.340	0	1	2,317
Female	0.319	0.466	0	1	2,317
No school education	0.377	0.485	0	1	2,317
Primary education	0.482	0.500	0	1	2,317
Secondary education	0.121	0.327	0	1	2,317
University education	0.023	0.151	0	1	2,317
No monthly income	0.119	0.324	0	1	2,317
Monthly income < = 750 Birr	0.346	0.476	0	1	2,317
Monthly income >750–1500 Birr	0.266	0.442	0	1	2,317
Monthly income >1500–3000 Birr	0.185	0.388	0	1	2,317
Monthly income >3000–12000 Birr	0.079	0.270	0	1	2,317
Monthly income >12000 Birr	0.005	0.069	0	1	2,317
Covered by health insurance	0.342	0.474	0	1	2,317
Myself or family sick with COVID-19	0.048	0.214	0	1	2,317
Myself or family have chronic illness	0.092	0.289	0	1	2,317
No trust in national government	0.177	0.382	0	1	2,317
Low trust in national government	0.172	0.377	0	1	2,317
Moderate trust in national government	0.322	0.467	0	1	2,317
High trust in national government	0.262	0.440	0	1	2,317
Participated in voluntary work for the common good	0.343	0.475	0	1	2,317
Live in rural areas	0.775	0.418	0	1	2,317
Live in Addis	0.044	0.205	0	1	2,317
Live in other urban area	0.181	0.385	0	1	2,317

* Based on the question: *“If a vaccine for COVID-19 gets introduced*, *would you like to get it”* and three exclusive answer options–(1) no (2) yes, only for free and (3) yes, even if I have to pay.

** Binary variable takes on a value of 0 if person answered (1 no) and 1 if answered (2 yes, only for free) or (3 yes, even if I have to pay).

*** Takes on a value of 1 if the person answered (3 yes, even if I have to pay) and 0 if the person answered (2 yes, only for free).

Respondents that were not willing to take the vaccine in the first place (1, no) are not included here. The variable should not be interpreted as a willingness to pay variable as question is hypothetical and no monetary amount was specified.

**Table 2:** Willingness to take COVID-19 vaccine* by covariates.

Variable	Willingness to take COVID-19 vaccine**	Willingness to take COVID-19 vaccine even if having to pay for it[unspecified monetary amount and hypothetical]***COVID
	Mean	Mean
Age 15–29	0.91	0.38
Age 30–39	0.86	0.31
Age 40–49	0.86	0.28
Age 50–59	0.89	0.33
Age >60	0.87	0.35
Female	0.88	0.33
Male	0.88	0.32
No school education	0.87	0.29
Primary education	0.88	0.35
Secondary education	0.89	0.38
University education	0.89	0.42
No monthly income	0.85	0.27
Monthly income < = 750 Birr	0.91	0.26
Monthly income >750–1500 Birr	0.88	0.31
Monthly income >1500–3000 Birr	0.86	0.45
Monthly income >3000–12000 Birr	0.85	0.51
Monthly income >12000 Birr	0.92	0.60
Covered by health insurance	0.88	0.36
Not covered by health insurance	0.88	0.31
Myself or family sick with COVID-19	0.96	0.36
No COVID-19 sickness	0.87	0.33
Myself or family have chronic illness	0.87	0.32
No chronic illness	0.88	0.33
No trust in national government	0.80	0.30
Low trust in national government	0.89	0.35
Moderate trust in national government	0.90	0.36
High trust in national government	0.92	0.32
Participated in voluntary work for the common good	0.90	0.32
Not participated (common good)	0.87	0.33

* Based on the question: *“If a vaccine for COVID-19 gets introduced*, *would you like to get it”* and three exclusive answer options–(1) no (2) yes, only for free and (3) yes, even if I have to pay.

** Binary variable takes on a value of 0 if person answered (1 no) and 1 if answered (2 yes, only for free) or (3 yes, even if I have to pay).

*** Takes on a value of 1 if the person answered (3 yes, even if I have to pay) and 0 if the person answered (2 yes, only for free).

Respondents that were not willing to take the vaccine in the first place (1, no) are not included here. The variable should not be interpreted as a willingness to pay variable as question is hypothetical and no monetary amount was specified.

### Predictors of willingness to take COVID-19 vaccines

Fig 1 displays odds ratios (OR) with 95% confidence intervals (CI) stemming from logistic regression models of the willingness to get vaccinated as a function of 22 socio-demographic, economic, health, trust and residence variables. To enable comparisons, the specification follows a closely related study [1]. Detailed regression output is presented in S1 Table. Respondents aged 30–39 (OR = 0.65; 95% CI (0.43,0.96)) and 40–49 (OR = 0·61; 95% CI (0·40,0·93)) were less willing to take the vaccine compared to the youngest respondents (15–29 years; excluded category). With a case of COVID-19 in the family the odds were 2.68 times as large relative to a family with no such case (95% CI (1·08,6·68)). Respondents reporting low (OR = 2·02; 95% CI (1·38,2·96)), moderate (OR = 2·52; 95% CI (1·82,3·48)) or high (OR = 2·85; 95% CI (1·99,4·09)) trust in the national government all displayed a significantly higher willingness to take the vaccine compared to those with no trust (excluded category). Relatedly, amongst respondents who displayed pro-social behavior, as indicated by participation in voluntary work for the common good of their community, the odds were 1·37 times larger (95% CI (1·03,1·81)). All other predictor groups (gender, education, income, health insurance, chronic illness, urban residence) did not significantly predict vaccine willingness at the 5% level.

### Predictors of willingness to take COVID-19 vaccine even if having to pay for it [unspecified monetary amount and hypothetical

Fig 2 presents the corresponding odds ratios from a logistic regression model where the dependent variable was the respondent’s *willingness to take COVID-19 vaccine even if having to pay for it [unspecified monetary amount and hypothetical]* (see definition below Fig 2). 12% of the respondents are excluded from this analysis as they are not willing to take the COVID-19 vaccine in the first place (N = 2,036). As in the previous model, age and trust significantly predict willingness to take and pay for the vaccine. In addition, income is significantly associated with the indicator. There is a strong income gradient with odds ratios increasing steadily from a monthly income of >1500 Birr. The comparator category was no income. When a respondent fell in the income group >1500–3000 Birr, the odds were 2.29 times as large (95% CI (1·58,3·31)). Likewise, the income group >3000–12000 Birr is associated with odds which were 3·00 times as large (95% CI (1·90,4·73)). The odds increase further in the highest income bracket >12000 Birr (OR = 5·12; (95% CI (1·55,16·84)). Finally, health insurance coverage is positively and significantly associated with the indicator (OR = 1·40; 95% CI (1·14,1·72)). The remaining predictors are insignificant at the 5% level (for detailed estimation output see S2 Table).

## Discussion

An overwhelming majority of household heads (88%) were willing to take the vaccine and amongst those a third (33%) were hypothetically willing to pay an unspecified amount. The high acceptance rate, which is consistent with proportions needed to achieve herd immunity, is in marked contrast to the global average, and places it alongside countries with high willingness to take vaccines such as China and South Korea [1]. Globally, based on surveys conducted in 19 countries including two in SSA, the willingness rate was 71.5% (55% in Russia to 88.6% in China) [1]. A systematic review assessing acceptance rates of COVID-19 vaccination from surveys carried out in 33 countries showed acceptance rates ranging from 23.6% in Kuwait to 97% in Ecuador with an overall acceptance rate of about 70% [8]. In the case of eight European countries [4] willingness to take the vaccine ranged from 62% in France to 80% in Denmark with a mean of 73.9%, which is at the margins of the population proportion that needs to be vaccinated to ensure herd immunity [4]. In the case of SSA, based on interviews with a minimum of 1,000 respondents, the average willingness to take a vaccine in 15 countries was 79% with a low of 15% in the case of Cameroon [9, 11]. Consistent with the results presented here, at 94% and 98%, vaccine acceptance was highest in Ethiopia [9, 13, 14]. The high acceptance rate in Ethiopia may be attributed to recent and substantial investments by the government in local health infrastructure and successful community-based health promotion and health insurance campaigns, including immunization programs for children, spearheaded by health extension workers [33].

Regarding socio-economic correlates, there were no differences in willingness to take the vaccine between rural and urban areas, between male and female genders or across education levels. Focusing on willingness to take, we observed age-related patterns. However, unlike results for other countries [1] where age and willingness are positively correlated, in the Ethiopian case the elderly (50+) were just as likely to accept being vaccinated as compared to the youngest (<30) age group (91%). As the risk of a symptomatic COVID-19 infection increases with age [34], vaccine campaigns in the country may need to contend with slightly lower (86%) vaccine acceptance rates amongst those aged 30–49. One reason for this finding might be that the fear of getting infected by COVID-19 is decreasing with age [35]. Fear or concern of getting the illness is one of the key aspects in determining the willingness to take the COVID-19 vaccine [36].

Similar to the literature [1], we find that trust in government is strongly associated with the willingness to take the vaccine and can contribute to public compliance with recommended actions. Trusted sources of information and guidance are crucial to disease control [1, 13, 37]. Relatedly, vaccination has been likened to a social contract [38] and its success at the population level relies on a critical number of vaccinated individuals. In line with this, we documented a positive association between prior pro-social behavior and willingness to take the vaccine. Fostering and emphasizing the benefits of vaccination from a societal point of view and taking into account trust and pro-social behavior that are also attributes of social cohesion [39] may encourage larger uptake of COVID-19 vaccines [40].

While the effects of age and trust in government on the willingness to take the COVID-19 vaccine even if having to pay for it [unspecified monetary amount and hypothetical] parallel those for willingness to take, for other covariates, there are differences. One third of the individuals reported enrolment in a health insurance (in the form of a Community-Based Health Insurance, CBHI). CBHI members may display an elevated valuation of preventive health care [41], which may explain the association with this augmented willingness to take the vaccine variable. Although we control for income, it could also be an asset effect driven by a positive link between household wealth and health insurance status. Regardless, what is clear is that this indicator is strongly related to income and ranges from 26–27% for the lowest income group to 60% for the highest income group (Table 2). A majority of the sample (75%) lies in the three lowest income brackets.

It is important to note that our indicator is **not** a WTP indicator, which would require a more sophisticated measurement tool. So further evidence is needed. But these results could hint at the need for a free or subsidized COVID-19 vaccination program, at least, covering those in the lower income brackets. In addition, previous evidence suggests that initial, one-off subsidies for preventive health goods can boost long-run adoption via learning [42].

The results highlight both opportunities and challenges. There is little evidence of vaccine hesitancy. Trust in government and pro-social behavior may be readily leveraged as the country rolls out its COVID-19 vaccine campaign. However, there is a possibility that deteriorating trust in the government due to the recent internal conflicts will threaten the effectiveness of vaccine campaigns.

Reaching higher levels of vaccination coverage may require a public-sector driven free vaccination campaign. However, out-of-pocket expenditure for health care is not unusual in Ethiopia. In 2018, OOP accounted for 35% of total health expenditure [43]. As in the case of other successful health campaigns such as the CBHI, which is financed by voluntary premium payments [44, 45], the country may consider relying on domestic private resources to enhance vaccine coverage. Nevertheless, even with such an approach, a free vaccination campaign is likely to remain essential [46].

## Conclusion

The willingness to take the vaccine in Ethiopia is high and in marked contrast to the global average. Our results are only representative of our target population at the time of the survey, and further evidence from other time periods and LICs is needed to get a complete picture. At least in our context and at that time, there is a large gap between the willingness to take compared to the willingness to take and hypothetically pay (an unspecified amount) for a COVID-19 vaccine. While we did not explicitly measure WTP in this survey and thus further evidence is needed, one may argue that external funding for vaccination campaigns is required. In addition to building confidence, as suggested by association with trust in the government and pro-social behaviour, it seems that fairly-priced vaccines along with financial support are needed to ensure uptake of COVID-19 vaccines in Ethiopia. Policy makers should consider these factors in the planning of COVID-19 vaccine campaigns to ensure uptake of COVID-19 vaccines.

### Ethics approval and consent to participate

The survey was approved by all regional state offices in Ethiopia after the review of the provided guidelines for the study procedures including the questionnaire: (Afar ref. number: 02/42/02/603/13; Amhara ref. number: ANRG PC/Liyu-01/56; Oromia ref. number: 04/Dh-78/1/68; Tigray ref. number: A/S/395/Z/C/02/2013; SNNPR ref. number: 7/3-1-20-58/1387; Sidama ref. number: S/D/01M/L/D/H/192; Somali ref. number: XSHAIS68-43; Dire Dawa ref. number: 09-10-59-4-59; Addis Ababa ref. number: 024/32/12/503/11).

Ethics approval was provided by the Research Ethics Committee of the International Institute of Social Studies, Erasmus University Rotterdam.

The survey was realized as a joint project between the German Development Institute (Deutsches Institut für Entwicklungspolitik, DIE), Friedrich-Ebert-Stiftung (FES) and the International Labour Office (ILO). National survey institutes (NSIs) that are part of the AfroBarometer network were the implementing partners in the survey countries. Technical support, including data management, was provided by the Institute for Development Studies (IDS), University of Nairobi. Members of these institutions met on several occasions to jointly develop the questionnaire and agree on details of the survey protocol. The questionnaire was administered to gather information from respondents after obtaining their oral informed consent about the survey. No personally identifiable information was stored.

## Supporting information

S1 Table Willingness to take COVID-19 vaccine*. (DOCX)

S2 Table Willingness to take COVID-19 vaccine even if having to pay for it [unspecified monetary amount and hypothetical]*. (DOCX)

S1 File Sampling process. (DOCX)

S1 Questionnaire (DOCX)

S2 Questionnaire (PDF)
