# Supplementary material for: Willingness to take COVID-19 vaccination in low-income countries: Evidence from Ethiopia
Source: PLoS One. 2022 Mar 3;17(3):e0264633. doi: 10.1371/journal.pone.0264633 (PMC8893640; doi:10.1371/journal.pone.0264633)
Supplement: S1 Table — (DOCX) [file pone.0264633.s001.docx]

**S1 Table. Willingness to take COVID-19 vaccine***

|  | Odds Ratio | Std. Err. | Z | P>\|Z\| | [95% Conf. Interval] | |
| --- | --- | --- | --- | --- | --- | --- |
| Age 30-39 [reference category: age 15-29] | 0.65 | 0.13 | -2.15 | 0.032 | 0.43 | 0.96 |
| Age 40-49 | 0.61 | 0.13 | -2.29 | 0.022 | 0.40 | 0.93 |
| Age 50-59 | 0.92 | 0.24 | -0.33 | 0.743 | 0.55 | 1.53 |
| Age >60 | 0.77 | 0.20 | -1.04 | 0.299 | 0.47 | 1.26 |
| Female | 1.01 | 0.15 | 0.08 | 0.937 | 0.76 | 1.36 |
| Primary education [reference category: no education] | 1.09 | 0.16 | 0.56 | 0.579 | 0.81 | 1.46 |
| Secondary education | 1.15 | 0.30 | 0.54 | 0.592 | 0.69 | 1.92 |
| University education | 1.09 | 0.56 | 0.16 | 0.870 | 0.40 | 2.96 |
| Monthly income <=750 Birr [reference category: no income] | 1.33 | 0.31 | 1.21 | 0.226 | 0.81 | 2.11 |
| Monthly income >750-1500 Birr | 0.78 | 0.18 | -1.06 | 0.288 | 0.50 | 1.23 |
| Monthly income >1500-3000 Birr | 0.67 | 0.16 | -1.64 | 0.100 | 0.42 | 1.08 |
| Monthly income >3000-12000 Birr | 0.80 | 0.26 | -0.69 | 0.489 | 0.43 | 1.50 |
| Monthly income >12000 Birr | 1.45 | 1.57 | 0.35 | 0.728 | 0.18 | 12.02 |
| Covered by health insurance | 1.00 | 0.14 | -0.03 | 0.980 | 0.76 | 1.31 |
| Myself or family sick with COVID-19 | 2.68 | 1.25 | 2.12 | 0.034 | 1.08 | 6.68 |
| Myself or family have chronic illness | 0.93 | 0.21 | -0.31 | 0.757 | 0.60 | 1.45 |
| Low trust in government [reference category: no trust in gov.] | 2.02 | 0.39 | 3.64 | 0.000 | 1.38 | 2.96 |
| Moderate trust in government | 2.52 | 0.42 | 5.57 | 0.000 | 1.82 | 3.48 |
| High trust in government | 2.85 | 0.53 | 5.69 | 0.000 | 1.99 | 4.09 |
| Participated in voluntary work for the common good | 1.37 | 0.20 | 2.18 | 0.029 | 1.03 | 1.81 |
| Live in Addis [reference category: Live in rural areas] | 0.89 | 0.32 | -0.34 | 0.732 | 0.44 | 1.78 |
| Live in other urban area | 0.83 | 0.15 | -1.06 | 0.290 | 0.58 | 1.18 |
| Number of observations | 2,317 |  |  |  |  |  |

Note: Logistic regression model. * Based on the question: *“If a vaccine for COVID-19 gets introduced, would you like to get it”* and three exclusive answer options – (1) no (2) yes, only for free and (3) yes, even if I have to pay. Binary variable takes on a value of 0 if person answered (1 no) and 1 if answered (2 yes, only for free) or (3 yes, even if I have to pay).
