# Supplementary material for: Willingness to take COVID-19 vaccination in low-income countries: Evidence from Ethiopia
Source: PLoS One. 2022 Mar 3;17(3):e0264633. doi: 10.1371/journal.pone.0264633 (PMC8893640; doi:10.1371/journal.pone.0264633)
Supplement: S2 Table — (DOCX) [file pone.0264633.s002.docx]

**S2 Table. Willingness to take COVID-19 vaccine even if having to pay for it [unspecified monetary amount and hypothetical]***

|  | Odds Ratio | Std. Err. | Z | | P>\|Z\| | | [95% Conf. Interval] | | |
| --- | --- | --- | --- | --- | --- | --- | --- | --- | --- |
| Age 30-39 [reference category: age 15-29] | 0.73 | 0.10 | -2.28 | | 0.022 | | 0.55 | | 0.96 |
| Age 40-49 | 0.64 | 0.10 | -2.85 | | 0.004 | | 0.48 | | 0.87 |
| Age 50-59 | 0.90 | 0.16 | -0.63 | | 0.532 | | 0.64 | | 1.26 |
| Age >60 | 1.04 | 0.18 | 0.22 | | 0.828 | | 0.74 | | 1.46 |
| Female | 1.21 | 0.14 | 1.69 | | 0.092 | | 0.97 | | 1.50 |
| Primary education [reference category: no education] | 1.24 | 0.14 | 1.84 | | 0.065 | | 0.99 | | 1.55 |
| Secondary education | 1.16 | 0.22 | 0.78 | | 0.438 | | 0.80 | | 1.68 |
| University education | 1.00 | 0.35 | 0.01 | | 0.989 | | 0.51 | | 1.99 |
| Monthly income <=750 Birr [reference category: no income] | 0.93 | 0.16 | -0.42 | | 0.672 | | 0.66 | | 1.31 |
| Monthly income >750-1500 Birr | 1.25 | 0.22 | 1.24 | | 0.216 | | 0.88 | | 1.77 |
| Monthly income >1500-3000 Birr | 2.29 | 0.43 | 4.41 | | 0.000 | | 1.58 | | 3.31 |
| Monthly income >3000-12000 Birr | 3.00 | 0.70 | 4.72 | | 0.000 | | 1.90 | | 4.73 |
| Monthly income >12000 Birr | 5.12 | 3.11 | 2.69 | | 0.007 | | 1.55 | | 16.84 |
| Covered by health insurance | 1.40 | 0.15 | 3.26 | | 0.001 | | 1.14 | | 1.72 |
| Myself or family sick with COVID-19 | 1.15 | 0.26 | 0.64 | | 0.523 | | 0.75 | | 1.78 |
| Myself or family have chronic illness | 0.94 | 0.16 | -0.35 | | 0.723 | | 0.67 | | 1.32 |
| Low trust in government [reference category: no trust in gov.] | 1.39 | 0.22 | 2.08 | | 0.038 | | 1.02 | | 1.90 |
| Moderate trust in government | 1.51 | 0.21 | 2.97 | | 0.003 | | 1.15 | | 1.97 |
| High trust in government | 1.28 | 0.19 | 1.68 | | 0.093 | | 0.96 | | 1.70 |
| Participated in voluntary work for the common good | 0.93 | 0.10 | -0.67 | | 0.504 | | 0.76 | | 1.15 |
| Live in Addis [reference category: Live in rural areas] | 0.66 | 0.17 | -1.58 | | 0.114 | | 0.40 | | 1.10 |
| Live in other urban area | 0.92 | 0.12 | -0.64 | | 0.524 | | 0.70 | | 1.20 |
| Number of observations | 2,036 |  | |  | |  | |  |  |

Note: Logistic regression model. * Based on the question: *“If a vaccine for COVID-19 gets introduced, would you like to get it”* and three exclusive answer options – (1) no (2) yes, only for free and (3) yes, even if I have to pay. Binary variable takes on a value of 1 if the person answered (3 yes, even if I have to pay) and 0 if the person answered (2 yes, only for free). Respondents that were not willing to take the vaccine in the first place (1, no) are not included here. The variable should not be interpreted as a willingness to pay variable as question is hypothetical and no monetary amount was specified.
