# Supplementary material for: Willingness to take COVID-19 vaccination in low-income countries: Evidence from Ethiopia
Source: PLoS One. 2022 Mar 3;17(3):e0264633. doi: 10.1371/journal.pone.0264633 (PMC8893640; doi:10.1371/journal.pone.0264633)
Supplement: S1 File — (DOCX) [file pone.0264633.s003.docx]

The sampling process was structured in four stages and follows the sampling process that is applied in the Afrobarometer surveys [1]: (i) selection of enumeration areas; (ii) selection of sampling start-points; (iii) selection of households; and (iv) selection of respondents for interview.

*i) Selecting enumeration areas (EA):* Based on the latest and updated population census the central statistics agency (CSA) in Ethiopia randomly select enumeration areas for each stratum^[[1]](#footnote-1)^ and respective rural/urban divide, based on probability proportional to size of population. In total 292 enumeration areas have been selected.

*ii) Selecting the sampling start-points (SSPs) for each enumeration area:* As no complete lists of households of the informal economy were available from which the sample could be randomly drawn for each EA, we use physical maps of the enumeration areas that were provided by the CSA in Ethiopian. A random sampling start-point (SSP) is marked on the map and field teams travel as close as possible to it, or to housing settlements nearest to it. A second SSP is selected as a reserve or substitute in case the initial SSP is inappropriate or inaccessible. Random selection of a start-point uses a grid. A ruler is placed along the top of the map and another along the side. A table of random numbers is then used to select pairs of numbers, one for the top axis and one for the side axis, resulting in a random combination. A line is then drawn on the map horizontal to the number chosen on the side, and another line is drawn vertical to the number chosen on the top. The point on the map where these two lines intersect is the sampling start-point. Each x-Y pair of numbers from the random number table can be used only once.

*iii) Selecting the household – walking pattern of interview teams:* The interviewers start walking away from the physical start-point, with interviewer 1 walking towards the sun; interviewer 2 in the opposite direction; interviewers 3 and 4 at a 90-degree angle to the right and left. With this walking pattern, all four directions are covered. By counting households on both sides of the walking path, household No. 5 is selected as the first household for the interview and household No. 15 for the second interview. Special rules were applied in the case of multi-storey buildings, widely scattered households and settlements within commercial farms.

If the interview cannot take place because nobody is at home, or the interview starts but cannot be finished, the walk continues to the next household on the same side of the road or opposite (household No. 6), while the second interview is done in household No. 16.

If the interview is refused the walk continues in the same direction until household No. 15. The second interview would take place with household No. 25.

*iv) Identifying respondents for the interview that work in the informal economy*: At the household level the interview is conducted with the household head living in the household. If the household head is unavailable the enumerator made an appointment for a later time in the day for a second attempt. If the interview is unsuccessful after the second attempt, the enumerator selected another respondent that is most knowledgeable about all the other members of the household. If the second respondent is unavailable or the interview is unsuccessful for whatever reason, the household is dropped and the enumerator replaces it with another household.

The household head or most knowledgeable person had to provide first information on her/his working status and on each member of the household (15 or older). The interview was ended if the household head is not active in the informal economy and the household was replaced by another household. The interview was continued if the household head or the most knowledgeable person is part of the informal economy.

To identify activities within the informal economy, the survey used the following operational definitions: i) Informal farming, raising animals or fishing: economic activities whose products have been produced for sale were grouped as informal. ii) Informal employees: paid job with reference to an employer’s tax contribution or contribution to a public or private pension scheme. If employers did not pay contributions, employees were grouped as informal. iii) Informal employers and own-account workers: informality is defined by non-registration in the national registry, which is used for company taxation. iv) Contributing family workers: defined, by default, as having an informal job because of the informal nature of jobs held by contributing family workers.

**References**

1. Afrobarometer Survey Manual (2017)

https://afrobarometer.org/sites/default/files/survey_manuals/ab_r7_survey_manual_en1.pdf

1. [↑](#footnote-ref-1)
