# Supplementary material for: Willingness to take COVID-19 vaccination in low-income countries: Evidence from Ethiopia
Source: PLoS One. 2022 Mar 3;17(3):e0264633. doi: 10.1371/journal.pone.0264633 (PMC8893640; doi:10.1371/journal.pone.0264633)
Supplement: S2 Questionnaire — (PDF) [file pone.0264633.s005.pdf]

INFORMAL ECONOMY, SOCIAL PROTECTION AND POLITICAL TRUST IN ETHIOPIA

-Questionnaire-

Content

Block 1: Identification and Basic Information ... 3

Block 2a: Household screening incl. health status ... 6

Block 2b: Household screening: employment status ... 9

Block 3a: Household information ... 21

Block 3b: Use of health services ... 28

Block 3c: COVID-19 ... 30

Block 4a: Basic information about selected household member ... 36

Block 4b: Informal employment – Main job of selected household member ... 39

Block 5: Citizen-state relationship of selected household member ... 42

Block 6: Use of health services by selected household member ... 50

Block 7a: Participation in CSOs ... 54

Block 7b: Views on Trade Unions ... 60

Block 8: Closing information [to be filled by the interviewer] ... 64

Block 1: Identification and Basic Information

**B1 Q01. Fieldworker No.**				

**B1 Q02. PSU/EA**: *[Circle one]*	
Urban	1
Rural	2

*[**Interviewer**: Select appropriate code for Region. Write names for County, District and Town / Village and EA number in the boxes.]*			
**B1 Q03. Region**		**B1 Q04.Province**	
		**B1 Q05. District**	
		**B1 Q06. Town/Village**	
		**B1 Q07. EA Number**	

**B1 Q08. GPS COORDINATES**	**Latitude**	
	**Longitude**	

**B1 Q09.** *[**Interviewer**: Was the last call you made successful?*	
Yes	1
No	0

If **B1 Q09. ==0 go to B1 Q10 (This can be a loop _Code as B1_Q1A.... B1_Q1B... Follow guidelines above. After any interview ends this must start again at B1_Q1A to reconstruct how many nocall happened before an interview)**

**B1 Q10.** *[**Interviewer**: Please code the information on tried interviews that did not work*							
**NOCALL**	**NOCALL1**	**NOCALL2**	**NOCALL3**	**NOCALL4**	**NOCALL5**	**NOCALL6**	**NOCALL7**
**Reasons for Unsuccessful Calls**	**HH 1**	**HH 2**	**HH 3**	**HH 4**	**HH 5**	**HH 6**	**HH 7**
Refused to be interviewed	1	1	1	1	1	1	1
Household head/knowledgeable member was not at home	2	2	2	2	2	2	2
First selected person was never at home after at least after two visits	3	3	3	3	3	3	3
Second selected person was not at home	4	4	4	4	4	4	4
Not a citizen / Spoke only a foreign language	5	5	5	5	5	5	5
Deaf / Did not speak a survey language	6	6	6	6	6	6	6
No one in the household belongs to informal sector	7	7	7	7	7	7	7
Did not fit gender quota	8	8	8	8	8	8	8
Other *[Specify]*____________________________	9	9	9	9	9	9	9
Not applicable	97	97	97	97	97	97	97

When you find a household with someone at home, please introduce yourself using the following script. You must learn this introduction so that you can say it exactly as it is written below.

Consent form:

Good day. My name is ____________. I am from ________ an independent research organization. We are conducting a study in cooperation with Friedrich Ebert Stiftung, the International Labour Organisation and the German Development Institute. I do not represent the government or any political party. We are studying the situation of citizens in Ethiopia operating in the informal economy. Every person of the informal economy has an equal chance of being included in this study. All information will be kept confidential. Your household has been chosen by chance.

We would like to discuss the issues at hand with members of your household. Actually the interview has two broad parts. For the first part, we would like to talk to the **head of the household**. For a second part, a second person (potentially again the head of the household) would be selected by chance.

Is the head of the household available and willing to talk to me and contribute to this study?

[**Interviewer**: If you did not talk with the head of the household first, please repeat introduction when meeting him/her.

Good day. My name is ____________. I am from ________ an independent research organization. We are conducting a study in cooperation with Friedrich Ebert Stiftung, the International Labour Organisation and the German Development Institute. I do not represent the government or any political party. We are studying the situation of citizens in Ethiopia operating in the informal economy. Every person of the informal economy has an equal chance of being included in this study. All information will be kept confidential. Your household has been chosen by chance.

We would like to discuss the issues at hand with members of your household. Actually the interview has two broad parts. For the first part, we would like to talk to the head of the household. For a second part, a second person (potentially again the head of the household) would be selected by chance.

The answers will be confidential. They will be put together with 2336 other people we are talking to, to get an overall picture. It will be impossible to pick you out from what you say, so please feel free to tell us what you think. This interview will take about 60 minutes. There is no penalty for refusing to participate. Do you wish to proceed?

[**Interviewer**: Just as a reminder- the second part of the survey will be conducted with a randomly selected person from among a list of qualified people. In case that second part of the interview is done with a person you have not yet talked with, pls. remember to always repeat the introduction again.]

Good day. My name is ____________. I am from ________ an independent research organization. We are conducting a study in cooperation with Friedrich Ebert Stiftung, the International Labour Organisation and the German Development Institute. I do not represent the government or any political party. We are studying the situation of citizens in Ethiopia operating in the informal economy. Every person of the informal economy has an equal chance of being included in this study. All information will be kept confidential. Your household has been chosen by chance.

Within your household you were also selected by chance. Your answers will be confidential. They will be put together with 2336 other people we are talking to, to get an overall picture. It will be impossible to pick you out from what you say, so please feel free to tell us what you think. This interview will take about 50 minutes. There is no penalty for refusing to participate. Do you wish to proceed?

[Interviewer: proceed with interview only if answer is positive]

[Interviewer: establish respondent’s preferred language for interview (English or translated language) and other details listed below]

**B1 Q11. . DATEINTR**	Day		Month		Year	
Date of interview *[Interviewer: Enter day, month, and year]*						

**B1 Q12. STRTIME**	Hour		Minute	
Time interview started *[Interviewer: Enter hour and minute, use 24 hr. cloc*				

Block 2a: Household screening incl. health status

Basic Information about the block:

This block is asked the most knowledgeable person in the household. The most knowledgeable person in the household should answer about all members (including all children) of the household.

[The easiest way to think about this is a loop that you have to collect information for every member of the household. At the end of each loop please ask if there is another person that should be considered.]

Introduction:

Thank you for contributing to this study. First we would like to know a bit about the activities and health coverage of all the members of your household (including all children). Could you give us the name of the different persons and then respectively his/her gender, their relationship to you and whether they are covered by an insurance. We can go through the list step by step. Maybe the easiest is starting with yourself.

**Q1. How many persons (including you) are living in the household?**	

**Q2. Could you tell me your first and last name?_______**

**Q3. What is your age?**	_	_	_

**Q4. Gender (to be filled by the interviewer)**	
Male	1
Female	2

**Q5. What is your relationship to the household head?** *[Do not read options]*	
I'm household head	1
Spouse/partner	2
Son/daughter	3
Mother/father	4
Other relative	5
Domestic worker	6
Other unrelated person	7

**Q6. Are you covered by any health insurance?** *[If there are more than one health insurance scheme, please ask for the main one. Main health insurance is the one that have been used mostly.*	
Yes, public health scheme such as the CBHI (Community- based health insurance scheme)	1
Yes, private health insurance	2
Yes, micro-insurance scheme	3
Previously had health insurance but dropped out	4
Never had health insurance	5
Do not know *[Do not read]*	9

**FILTER:**
**ASK ONLY IF Q6=1 OR Q6=2 OR Q6=3. OTHERWISE GO TO Q9**

**Q7. Are you covered on a voluntary or mandatory basis?**	
Mandatory	1
Voluntary	2
Don't know *[Do not read]*	9

**FILTER:**
**SEE FILTER ABOVE: THIS QUESTION SHOULD ONLY BE ASKED IF Q6=1 OR Q6=2 OR Q6=3**

**Q8. Are you /your employer the main contributor to the scheme or is it covered indirectly through another family member?**	
I am the main contributor	1
Another family member is the main contributor	2
My employer is the main contributor	3
Don't know *[Do not read]*	9

Thank you for these insights. Now I would like to know similar information on all members of the household. Could you give us the names of the different persons and then respectively his/her gender, their relationship to you and whether they are covered by an insurance The easiest is going for the question for each person individually

[Here a loop starts! It has to go for as many persons as the person in the household lists _ The coding of the question should change with each additional person Q9x (with x = 9a; 9b; 9c; …]

**Q9x. Could you tell me the first name and the last name of another person living in your household____________**

**Q10x. What is NAME's age?**	_	_	_

**Q11x. What is NAME's gender?**	
Male	1
Female	2

Q12x. What is NAME's relationship to the head of the household? *[Do not read options]*	
Household head	1
Spouse/partner	2
Son/daughter	3
Mother/father	4
Other relative	5
Domestic worker	6
Other unrelated person	7

Q13x. Is NAME covered by any health insurance? *[If there are more than one health insurance scheme, please ask for the main one. Main health insurance is the one that have been used mostly.]*	
Yes, public health scheme such as CBHI (Community-based health insurance scheme)	1
Yes, private health insurance	2
Yes, micro-insurance scheme	3
No, ------------ had health insurance previous, but dripped out	4
No, had never a health insurance	5
I don't know	9

FILTER:
ASK ONLY IF Q5=1 OR Q5=2 OR Q5=3 OTHERWISE GO TO Q9 (next member of household)

Q14x. Is NAME covered on a voluntary or mandatory basis?	
Mandatory	1
Voluntary	2
Don't know *[Do not read]*	9

FILTER:
SEE FILTER ABOVE: THIS QUESTION SHOULD ONLY BE ASKED IF Q5=1 OR Q5=2 OR Q5=3

Q15x. Is NAME the main contributor to the scheme or is it covered indirectly through another family member	
He/she is the main contributor	1
Another household member (including the household head) is the main contributor	2
My/his/her employer is the main contributor	3
Don't know *[Do not read]*	9

Q16x. Is there another member of your household we have not talked about yet? *[Do not read options]*		
Yes	1	Go to / Q9x [start the loop again]
No	2	Go to Block B2b [Question / Q17.]

Block 2b: Household screening: employment status

[The easiest way to think about this is a loop that you have to make for every member of the household declared that is 15 years old or above and where the relationship with the household head is not labelled as being 6 = “Domestic worker”)]

Basic Information about the block:

This block is asked the most knowledgeable person in the household. The most knowledgeable person should answer about all members of the household 15 years or older, individually. In the case, where the relationship to the most knowledgeable person is “domestic worker”, we do not need the information. In the case, the most knowledgeable person is a domestic worker, we do not need the information.

FILTER: / **Q3. <15 OR Q5 = 6** ***Most knowledgeable person SHOULD NOT BE CONSIDERED***

FILTER: / **Q10a-z <15 ou Q12a-z=6** ***PERSON SHOULD NOT BE CONSIDERED***

Now I will ask you about the main activity that you and the members of your household spend much of your time on. I will also like to know some details connected to this. We will proceed as before. It would need you to share the information on each member of the household. Let’s start with you.

**Q17. Which of the following activities best describes what YOU are MAINLY doing at present?**	
Working in a farm, raising animals or fishing	1
Working in another activity (outside agriculture/Fishery)	2
Studying	3
Unemployed - Looking for work	4
Taking care of household or family member	5
Not working - with long-term illness or disability	6
Not working - retired or pensioner	7
Other	8

**FILTER: ASK ONLY IF Q17=1**		
**Q18. Are these products intended mainly for sale or for family consumption?**		
Only for sale	1	
Mainly for sale	2	
Mainly for family consumption	3	GO TO QAH1 (between Q24 and Q25)
Only for family consumption	4	GO TO QAH1 (between Q24 and Q25)

**FILTER:**
**ASK ONLY IF Q17>2 (i.e. Q17=3 OR Q17=4 OR Q17=5 OR Q17=6 OR Q17=7 OR Q17=8). =**

**Q19.1. During the last four weeks did YOU do any work FOR SOMEONE ELSE for pay, even if only for one hour?**	
YES	1
No	2

**FILTER:**
**ASK ONLY IF Q19.1=2**

**Q19.2. During the last four weeks did YOU run or do ANY KIND of business, farming or other activity to generate income, even if only for one hour?**	
YES	1
NO	2

**FILTER: ASK ONLY IF Q19.2=2**

**Q19.3. During the last four weeks, did YOU help with the paid job or business of a family member?**	
YES	1
NO	2

**FILTER: ASK ONLY IF Q17=2 OR (Q17=1 AND (Q18=1 OR Q18=2)) OR (Q17>2 AND Q19.1=1 OR Q19.2=1 or Q19.3=1)**

**Q20. During the last four weeks did (YOU) have more than one job/business?**	
Yes, One job/business	1
Yes, More than one job/business	2

**FILTER:**
**READ TEXT ONLY IF Q20=2**
**TEXT:** I am now going to ask you some questions about YOUR main job. The main job is the one where YOU usually work the highest number of hours even if YOU were temporarily absent.

**FILTER:**
**ASK ONLY IF Q17=2 OR (Q17=1 AND (Q18=1 OR Q18=2)) OR (Q17>2 AND Q19.1=1 OR Q19.2=1 or Q19.3=1)).**

**Q21. In your main job do YOU work…?**<br><br>*[Note for interviewer: If person is unemployed, studying, taking care of family members but has generated an income or helped in a family business during the last four weeks* ***ask about their last work situation****.]*	
In (your/his/her) own business activity	1
As an employee for someone else	2
As an apprentice, intern	3
In a business operated by a household or family member	4

**FILTER:**
**ASK ONLY IF Q21=1 OTHERWISE MOVE TO Q24**

**Q22. Do you have regular employees?**	
Yes	1
No	2

**FILTER:**
**SEE FILTER ABOVE: THIS QUESTION SHOULD ONLY BE ASKED IF Q21=1**

**Q23. Is YOUR business registered in the National register of companies?**	
Yes	1
No	2
Don't know *[Do not read]*	9

**FILTER:**
**ASK ONLY IF (Q21=2) OR (Q21=3) OR (Q21=4).**

**Q24. Does YOUR employer pay contributions to YOUR**	
Public pension	1
Private Pension	2
No pension	3
Don´t know *[Do not read]*	9

IF Q17=2,3,4,5,6,7,8 then text: **Thanks for that information. Let's talk about other members in your household. What about /NAME?**

IF Q17=1 then enter adhoc module on additional activities for subsistence workers (i.e QAH1)

Ad-hoc module additional activities for subsistence workers

FILTER:
ASK ONLY IF Q17=1

QAH1. During the last year, did you do any work for pay in addition to your work in the farm, raising animals or fishing?	
YES	1
No	2

FILTER:
ASK ONLY IF QAH1=1 OTHERWISE MOVE TO Q25x

QAH2. Was any of this work carried out …<br>(READ ALL, MARK ALL THAT APPLY)	
for a family member (for pay) in agriculture or other business	1
As an additional own business activity (e.g. selling self-made non-agriculture products, handicraft, kiosk …)	2
As an employee for someone else	3

FILTER:
ASK ONLY IF QAH2=3

QAH3. During the last year, have you worked for one employer only or several employers?	
One employer only	1
Worked for more than one employer	2

FILTER:
ASK ONLY IF If QAH2=3 and QAH3=1

QAH4.A How many persons including you, worked for this employer?	
1 person (only me)	1
2-10 persons	2
11-50 persons	3
51 and more persons	4

FILTER:
ASK ONLY IF If QAH2=3 and QAH3=2

QAH4.B If you think about the employer for which you have carried out most work, how many persons including you worked for this employer?	
1 person (only me)	1
2-10 persons	2
11-50 persons	3
51 and more persons	4

FILTER:
ASK ONLY IF QAH1=1

QAH5. If you think about all the activities that you have carried out for pay during the last year and that is outside your own farm, fishing, raising animals, how much income do you think this has generated?

Range (in BIRR)		
From	To	
3.000 [250 / month]	6.000	1
6.000 [500 / month]	12.000	2
12.000 [1.000 / month]	18.000	3
18.000 [1.500 / month]	24.000	4
24.000 [2.000 / month]	30.000	5
30.000 [2.500 / month]	36.000	6
More than 36.000 [3.000 / month]		7

FILTER:
ASK ONLY IF QAH1=1

**QAH6. Considering this additional income, how important for the well-being / survival of your family was this part of your income?**	
**Very important**<br>(We could NOT survive without it (not enough food, would lose shelter, …)	1
**Important**<br>(we could BARELY survive without it (e.g. not buy new essential household goods, new cloth / shoes)	2
**Somewhat important**<br>(we could survive, but not pay school fees, health related costs and the like)	3
**Less important**<br>(We used the pay only for buying not so important things or for savings)	4

FILTER:
ASK ONLY IF QAH1=1

**QAH7 If you think about the year to come, what would happen if this additional income would be absent?**	
**Very large impact**<br>(we could NOT survive without it (not enough food, would loose shelter, …)	1
**Large impact**<br>(we could BARELY survive without it (e.g. not buy new essential household goods, new cloth)	2
**Some impact**<br>(we could survive, but not pay schools fees, for health-related costs)	3
**Little impact**<br>(we could live without it, but would feel the difference as we could not buy non-essential items, could not safe any money)	4

FILTER:
ASK ONLY IF QAH1=1

**QAH8 If you consider only the MAIN work you did outside your own farm: To which sector did this work belong?**	
Agriculture, forestry, fishing	1

Mining and quarrying	2
Manufacturing	3
Construction	4
Transport	5
Trade	6
Services (education, health, social & entertainment)	7
Other sector: pls. explain	8

**FILTER:**
**ASK ONLY IF QAH1=1**

**QAH9 If you look only at the additional work you have done outside your own farm: Can you give us an estimate of the duration of this work?**	
Less than one month	1
1-2 month	2
3-4 month	3
5-7 month	4
8-10 month	5
11-12 month / almost the whole year	6

**FILTER:**
**ASK ONLY IF QAH1=1**

**QAH10 If you consider the additional income: Did you invest part of it into your farm?**	
I did not use any money for my own farm	1
I used the smaller part of it for my own farm	2
I used the larger part of it for my own farm	3

**FILTER:**
**ASK ONLY IF QAH1=1 and QAH10=2 or 3**

**QAH11 What was the MAIN reason for you to spent money for your farm?**	
I bought farm inputs like seeds, plants or fertilizer	1
I bought equipment	2
I bought additional (plots of) land	3
I paid money to others who helped me with my work on the farm	4
I paid back money which I had borrowed previously for investments related to my farm	5
Other reason:¨pls. explain	6

Thanks for that information. Let's talk about other members in your household. What about /NAME?

[here is where the loop start: **Q25x with x= a, b…z**

**Q25x. Which of the following activities best describes what (/NAME) are MAINLY doing at present?**	
Working in a farm, raising animals or fishing	1
Working in another activity (outside agriculture/Fishery)	2
Studying	3
Unemployed - Looking for work	4
Taking care of household or family member	5
Not working - with long-term illness or disability	6
Not working - retired or pensioner	7
Other	8

FILTER: ASK ONLY IF Q25x=1

**Q26x. Are these products intended mainly for sale or for family consumption?**		
Only for sale	1	
Mainly for sale	2	
Mainly for family consumption	3	GO TO **QAH11x (between Q32 and Q33)**
Only for family consumption	4	GO TO **QAH11x (between Q32 and Q33)**

FILTER:
ASK ONLY IF Q25x>2 (i.e. Q25x=3 OR Q25x=4 OR Q25x=5 OR Q25x=6 OR Q25x=7 OR Q25x=8).

**Q27x.1. During the last four weeks did (NAME) do any work FOR SOMEONE ELSE for pay, even if only for one hour?**	
YES	1
No	2

FILTER:
ASK ONLY IF Q27x.1=2

**Q27x.2. During the last four weeks did (NAME) run or do ANY KIND of business, farming or other activity to generate income, even if only for one hour?**	
YES	1
NO	2

FILTER:
ASK ONLY IF Q27x.2=2

**Q27x.3. During the last four weeks, did (NAME) help with the paid job or business of a family member?**	
YES	1
NO	2

**FILTER:**
**ASK ONLY IF Q25x=2 OR (Q25x=1 AND (Q26x=1 OR Q26x=2)) OR (Q25x>2 AND Q27x.1=1 OR Q27x.2=1 or Q27x.3=1).**

**Q28x. During the last four weeks did (NAME) have more than one job/business?**	
One job/business	1
More than one job/business	2

**FILTER:**
**READ TEXT ONLY IF Q28x=2**

TEXT I am now going to ask you some questions about (/NAME)'s main job. The main job is the one where (/NAME) usually work the highest number of hours even if (NAME) (/were) temporarily absent.

**FILTER:**
**ASK ONLY IF Q25x=2 OR (Q25x=1 AND (Q26x=1 OR Q26x=2)) OR (Q25x>2 AND (Q27x.1=1 OR Q27x.2=1 or Q27x.3=1).**

**Q29x. In your main job does (/NAME) work…?**<br><br>*[Note for interviewer: If person is unemployed, studying, taking care of family members but has generated an income or helped in a family business during the last four weeks **ask about their last work situation**.]*	
In (your/his/her) own business activity	1
As an employee for someone else	2
As an apprentice, intern	3
In a business operated by a household or family member	4

**FILTER:**
**ASK ONLY IF Q29x=1 OTHERWISE MOVE TO / Q31x**

**Q30x. Does (/NAME) have regular employees?**	
Yes	1
No	2

**FILTER:**
**SEE FILTER ABOVE: THIS QUESTION SHOULD ONLY BE ASKED IF Q29x=1**

**Q31x. Is my/his/her business /farm registered in the National register of companies:**	
Yes	1
No	2
Don't know *[Do not read]*	9

**FILTER:**
**ASK ONLY IF Q29x=2) OR (Q29x=3) OR (Q29x=4).**

**Q32x. Does (/NAME's) employer pay contributions to**	
Public pension	1
Private Pension	2
No pension	3
Don´t know *[Do not read]*	9

IF Q25x =2,3,4,5,6,7,8 then text **Thanks for that information. What about /NAME?**

F Q Q25x =1 then enter adhoc module on additional activities for subsistence workers (i.e QAH11x)

Ad-hoc module additional activities for subsistence workers

**FILTER:**
**ASK ONLY IF Q25x=1**

**QAH12x. During the last year, does (/NAME) do any work for pay in addition to your work in the farm, raising animals or fishing?**	
YES	1
No	2

**FILTER:**
**ASK ONLY IF QAH12x=1 OTHERWISE MOVE TO NEXT HOUSEHOLD MEMBER OR NEXT SECTION**

**QAH13x. Was any of this work carried out …**<br>**(READ ALL, MARK ALL THAT APPLY)**	
for a family member (for pay) in agriculture or other business	1
As an additional own business activity (e.g. selling self-made non-agriculture products, handicraft, kiosk …)	2
As an employee for someone else	3

FILTER:
**ASK ONLY IF QAH13x=3**

**QAH14x. During the last year, has (/NAME) worked for one employer only or different employers?**	
One employer only	1
Worked for more than one employer	2

FILTER:
**ASK ONLY IF If QAH13x=3 and QAH14x=1**

**QAH15x.A How many persons including (/NAME), worked for this employer?**	
1 person (only me)	1
2-10 persons	2
11-50 persons	3
51 and more persons	4

FILTER:
**ASK ONLY IF If QAH13x=3 and QAH14x=2**

**QAH15x.B If you think about the employer for which (/NAME) have carried out most work, how many persons including (/NAME) worked for this employer?**	
1 person (only me)	1
2-10 persons	2
11-50 persons	3
51 and more persons	4

FILTER:
**ASK ONLY IF QAH12x=1**

QAH16x. If you think about all the activities that (/NAME) have carried out for pay during the last year and that is outside your own farm, fishing, raising animals, how much income do you think this has generated?

Range (in BIRR)		
From	To	
3.000 [250 / month]	6.000	1
6.000 [500 / month]	12.000	2
12.000 [1.000 / month]	18.000	3
18.000 [1.500 / month]	24.000	4
24.000 [2.000 / month]	30.000	5
30.000 [2.500 / month]	36.000	6
More than 36.000 [3.000 / month]		7

FILTER:
ASK ONLY IF QAH12x=1

QAH17x. Considering this additional income, how important for the well-being / survival of your family was the part of (/NAME`s) income?	
**Very important**<br>(We could NOT survive without it (not enough food, would lose shelter, …)	1
**Important**<br>(we could BARELY survive without it (e.g. not buy new essential household goods, new cloth)	2
**Somewhat important**<br>(we could survive, but not pay school fees, health related costs and the like)	3
**Less important**<br>(We used the pay only for buying not so important things or for savings)	4

FILTER:
ASK ONLY IF QAH12x=1

QAH18x If you think about the year to come, what would happen if this additional income would be absent?	
**Very large impact**<br>(we could NOT survive without it (not enough food, would loose shelter, …)	1
**Large impact**<br>(we could BARELY survive without it (e.g. not buy new essential household goods, new cloth)	2
**Some impact**<br>(we could survive, but not pay schools fees, for health-related costs)	3
**Little impact**<br>(we could live without it, but would feel the difference as we could not buy non-essential items, could not safe any money)	4

FILTER:
ASK ONLY IF QAH12x=1

QAH19x If you consider only the MAIN work (/NAME) did outside your own farm: To which sector did this work belong?	
Agriculture, forestry, fishing	1
Mining and quarrying	2
Manufacturing	3
Construction	4
Transport	5
Trade	6
Services (education, health, social & entertainment)	7
Other sector: pls. explain	8

FILTER:
ASK ONLY IF QAH12x=1

QAH20x If you look only at the additional work (/NAME) have done outside your own farm: Can you give us an estimate of the duration of this work?

Less than one month	1
1-2 month	2
3-4 month	3
5-7 month	4
8-10 month	5
11-12 month / almost the whole year	6

**TEXT Thanks for that information. What about /NAME?** **[here is where the loop ends; repeat as many times as necessary.]**
**Contributing family workers, employers and own-account workers in non-registered enterprises, apprentices, interns and employees without employer's contribution to social insurance should be set to IE=1 the rest should be set to IE=0**
**That is: 1:**
**1.1. FOR HH-HEAD OR HIS REPRESENTATIVE:** **IF (Q21=4) OR (Q21=1 AND Q23=2,9) OR (Q21=2, 3 AND Q24=3,9) THEN IE=1.**
**1.2. FOR OTHER HH-MEMBERS If (Q29x=4) OR (Q29x=1 AND Q31x=2,9) OR (Q29x=2,3 AND Q32x=3,9) THEN IE=1.**
**1.3. OTHERWISE IE=0**
**2: IF ALL HOUSEHOLD MEMBERS IE=0 THEN END OF INTERVIEW**
**3: RANDOMLY SELECT ONE PERSON WITH IE=1 AND CONTINUE THE INTERVIEW WITH THAT PERSON. THE PERSON WILL BECOME THE RESPONDENT FOR THE REST OF THE INTERVIEW AND WILL BE CODED IE=1**
**4: IF RANDOMLY SELECTED PERSON IS NOT AVAILABLE: MAKE AN APPOINTMENT AND VISIT THE HOUSEHOLD AGAIN**
**5: IF RANDOMLY SELECTED PERSON IS NOT AVAILABLE AT THE 2ND VISIT: RANDOMLY SELECT ANOTHER PERSON WITH IE=1**
**6: IF NEW RANDOMLY SELECTED PERSON IS NOT AVAILABLE: END THE INTERVIEW**

**ENDTIME**	Hour		Minute	
Time interview ended *[Interviewer: Enter hour and minute, use 24 hr. cloc*				

4: Indicate Line number of selected HH Member from B2a Q01 |_|_| in the respondent number

Block 3a: Household information

Basic Information about the block:
This block is asked the most knowledgeable person in the household= household head. The household head should answer about all member of the household. Starting here there will be no more loops

Q33. What is your highest level of education?
[Code from answer. Do not read options]

No formal schooling	0
Informal schooling only (including Koranic schooling)	1
Some primary schooling	2
Primary school completed	3
Junior secondary school or some secondary school / high school	4
Secondary school / high school completed	5
Post-secondary qualifications other than university, e.g. a diploma or degree from a polytechnic or college	6
Some university	7
University completed	8
Post-graduate	10
Don’t know *[Do not read]*	9

Q34. What is the roof of your shelter made of?
[Only ask this question when you cannot see the roof; Do not read options]

Metal, aluminium, tin, or zinc	1
Tiles	2
Shingles	3
Thatch or grass	4
Plastic sheets	5
Asbestos	6
Multiple materials	7
Concrete	8
Some other material	10
Multi-storey building	11

Q35. What is the wall of your shelter made of?
[Only ask this question when you cannot see the wall; Do not read options]

Mud	1
Bricks/Stones	2
Iron Sheet	3
Wood	4
Some other material	5
Multistorey building	6

Q36. What is the floor of your shelter made of?
[Only ask this question when you cannot see the roof; Do not read options]

Cement	1
Wood Floor	2
Earth	3
Some other material	4
Multistorey building	5

**Q37. Please tell me whether each of the following are available inside your house, inside your compound, or outside your compound:** *[Read out options]*						
		None, no latrine available *[DNR]*	Inside the house	Inside the compound	Outside the compound	Don't know *[DNR]*
A.	Your main source of water for household use?		1	2	3	9
B.	A toilet or latrine?	0	1	2	3	9

**Q38.** *Interviewer: If it is 100% clear that there is no electricity supply to the home, e.g. in an unserved rural area, do not ask this question. Just select 0=No electricity supply and continue to the next question.]* **Do you have an electric connection to your home from the mains?**	
No mains electric supply or connection to the home	0
[**If yes**] **How often is electricity actually available from this connection?**	
Never	1
Occasionally	2
About half of the time	3
Most of the time	4
All of the time	5
Don't know *[Do not read]*	9

[Thanks for all that information. Now we would like to know a bit more about the income in your household]

**Q39. Can you give me an estimate of your own average work related income and average household income in the last four weeks?** [Read out each options] [NOTE: after paying tax and after any contributions were deducted]				
		Range		
		From…	To…	
**1. Work related income (in BIRR)**	1	0	0	
	2	>0	750	
	3	>750	1500	
	4	>1500	3000	
	5	>3000	12,000	
	6	>12,000		
	8	Not willing		
	9	Don't	know	
**2. Average monthly household income (in BIRR)**	1	0	0	
	2	>0	750	
	3	>750	1500	
	4	>1500	3000	
	5	>3000	12,000	
	6	>12,000	50,000	
	7	>50,000		
	8	Not willing		
	9	Don't	know	

**Q40. Over the past 12 months, has your household's monthly income been regular, seasonal or irregular?** *[Interviewer: Probe for strength of opinion]*	
Very Irregular	1
Irregular	2
Seasonal	3
Regular	4
Very regular	5
Don't know *[Do not read]*	9

**Q41. Do you expect your household's income in the next 12 months to be higher, lower or about the same as compared to the current situation?**	
Lower	1
About the same	2
Higher	3
Don't know *[Do not read]*	9

**Q42. Over the last 12 months, what sources of finance have you used to finance large-scale expenses (exceeding your monthly income/revenue) in your household or business (Multiple answers are possible)?**	
Own savings or retained earnings	1
Friends and relatives	2
Moneylenders	3
Borrowed from private bank	4
Borrowed from state/public bank	5
Borrowed from cooperatives	6
Borrowed from microfinance institutions	7
Purchases on credit from suppliers and advances from customers	8
Other - Write In (Required): ____*	9
None	98

**Q43. How many of the following assets do you or someone in your household own?** *[Read out options]*	
	**Insert number of these assets in the household**
A. Generator	
B. Television	
C. Mobile telephone	
D. Non-mobile telephone	
E. Refrigerator	
F. Solar panel	
G. Table	
H. Chair	
I. Sofa	
J. Bed	
K. Cupboard	
L. Watch	

M. Microwave oven	
N. Radio	
O. Casette or CD Player	
P. Camera	
Q. Computer	
R. Bicycle	
S. Motorcycle or scooter	
T. Animal drawn cart	
U. Car or Truck	
V. Boat with a motor	

Q44. Over the past year, how often, if ever, have you or anyone in your family:
[Read out options]

	Never	Just once or twice	Several times	Many times	Always	Don't know *[DNR]*
A. Gone without enough food to eat?	0	1	2	3	4	9
B. Gone without enough clean water for home use?	0	1	2	3	4	9
C. Gone without medicines or medical treatment?	0	1	2	3	4	9
D. Gone without enough fuel to cook your food?	0	1	2	3	4	9
E. Gone without a cash income?	0	1	2	3	4	9

Read-out Text: "Many thanks for all this valuable information. Next, we would like to talk about how you and your household managed certain events."

	INT. Multiple answers		INT. Single answer
	Yes	No	**Which was the most serious event among those you have mentioned with regard to the expenses?**
**Q45A. During the last twelve months, has there been one or several of the events listed below that seriously affected your household's ability to pay the most essential expenses?**<br><br>[Health related shocks] [Read out options]			
A. Birth of a child (and pregnancy)	1	2	1
B. Hospitalization of income earning member	1	2	2
C. Hospitalization of other member of the family	1	2	3
D. Sickness due to COVID-19 virus infection	1	2	4
E. Other medical costs	1	2	5
F. Permanent disability	1	2	6
G. Temporary disability	1	2	7
H. Death of another member of the household	1	2	8
**Q46A. During the last twelve months, has there been one or several of the events listed below that seriously affected your household's ability to pay the most essential expenses?**<br><br>[Work related shocks: no or limited capacity to get an income from labour (yourself or from other HH members)] [Read out options]			
A. Do not have the opportunity to work	1	2	8
B. Do not have the opportunity to work anymore because of COVID-19 crisis	1	2	9
C. Loss of job/employment/work	1	2	10
D. Loss of job/employment/work because of COVID-19 crisis	1	2	11
E. Retirement from employment	1	2	12
F. Business failure	1	2	13
G. Business failure because of COVID-19 crisis	1	2	14
H. Death of income earning member	1	2	15
I. Harvest failure	1	2	16
**Q47A. During the last twelve months, has there been one or several of the events listed below that seriously affected your household's ability to pay the most essential expenses?**<br><br>[Other shocks] [Read out options]			
A. Natural disaster (drought, flood, fire etc)	1	2	14
B. Loss or destruction of property	1	2	15
C. Other (Specify) _____	1	2	16

FILTER:
ASK ONLY If Q45A.A-Q45A.G=1 OR Q46A.A–Q46A.F=1 OR Q47A.A–Q47A.C=1.

Q48. Which was the main coping strategy in case of 1. Health related shock; 2. Work related shock; 3. Other shock.
*[Do **not** read out options]*

		B3a Q14.1 **Health related** *[if yes in at least one the proposed answers among A to G in the previous]*	B3a Q14.2 **Work related** *[if yes in at least one the proposed answers among A to F in the previous]*	B3a Q14.3 **Other shocks** *[if yes in at least one the proposed answers among A to C in the previous]*
Spent savings		1	1	1
Sold machine, equipment or other asset used to generate income		2	2	2
Sold harvest in advance		3	3	3
Sold other assets		4	4	4
Took out mortgage on asset or house		5	5	5
Borrowed money from a bank		6	6	6
Borrowed money from money lenders		7	7	7
Borrowed money from other source		8	8	8
Worked longer hours		9	9	9
Sent children to work		10	10	10
Asked for support from state/ government /public entity		11	11	11
Asked for family (or friends) support	Living in the same country	12	12	12
	Living abroad	13	13	13
Asked for support from church, NGOs, or other actors than publics actors		14	14	14
Asked for support from cooperatives or professional associations		15	15	15
Other (Specify) __________		_________	_________	_________
Not applicable [do not read]		97	97	97

**Q49. In the last 12 months have you or has any member of you household received regular benefits in cash or in-kind? (Multiple answers possible)** [Read out options]			**IF YES: Q49.1 Who do you believe funded the benefit programme?**				
	Yes	No	Government/ State	Religious institution (Church)	International Institution (e.g.NGO, USAID)	Local NGO	DNK (Do not read)
A. Cash Transfer/ Direct-support from Productive Safety Net Programme (PSNP)	1	2	3	4	5	6	9
B. Food aid/ Community-Based Nutrition Programme (CBN)	1	2	3	4	5	6	9
C. Other social assistance	1	2	3	4	5	6	9
D. Cash for work/Productive Safety Net Programme (PSNP)	1	2	3	4	5	6	9
E. Food for work/Productive Safety Net Programme (PSNP)	1	2	3	4	5	6	9
F. Free medical services	1	2	3	4	5	6	9
G. Household Asset Building Programme (HABP)	1	2	3	4	5	6	9
H. In-School Feeding	1	2	3	4	5	6	9
I. Other regular payment in kind (specify………………)	1	2	3	4	5	6	9

**Q50. In the past 12 months, have you or your family assisted any other household in cash or in kind specifically because they experienced a health shock (need of money for medical treatment)?**	
Yes	1
No	2
Don´t know *[Do not read]*	9

**FILTER:**
**ASK ONLY If Q50 = 1 (YES). IF Q50= 2 OR 9 (NO or DNK) CONTINUE TO Q52.**

	**Amount**	Don't know	Not willing to respond
**Q51. If yes, please provide an estimate of the total value of the assistance in Ethiopian BIRR**	____________ ______	9	8

**Q52. In the past 12 months, have you received assistance from any other household in cash or in kind specifically because your household experienced a health shock (need of money for medical treatment)?**	
Yes	1
No	2
Don´t know *[Do not read]*	9

**FILTER:**
**ASK ONLY If Q52=1 (YES). If Q52 = 2 OR 9 (NO or DNK), CONTINUE TO NEXT Block**

	**Amount**	Refused to respond *[Do not read]*	Don't know *[Do not read]*
**Q53. If yes, please provide an estimate of the total value in Ethiopian BIRR (cash and in-kind) of the assistance your household received?**	______________ ____	8	9

Block 3b: Use of health services

Basic Information about the block:

This block is asked the most knowledgeable person in the household= household head. The household head should answer about all member of the household. Starting here there will be no more loops

Introduction:

We find it particularly relevant to understand how people use and perceive health services. In this line, the following questions focus on this aspect and we would appreciate you sharing your insights on this.

Q54. During the last 12 months, did you or any member of your household experience any of the following:

	Yes	No
A. … physical disability such as being blind, deaf, or limps deformation	1	2
B. … chronic illness (such as tuberculosis, HIV/AIDS, asthma, etc.)	1	2
C. … sickness due to COVID-19 virus infection	1	2
D. … pregnancies	1	2
E. … other health problem (such as accident, sickness etc.)	1	2

FILTER:
ASK ONLY IF Q54A=1 OR Q54B=1 OR Q54C=1 OR Q54D=1

**Q55. Over the last 12 months, during the last episode of illness in your family, what means of treatment did you seek?** *[Read out options] [Multiple responses possible]*	Yes	No
A. No treatment sought	1	2
B. Healer and traditional pharmacopoeia	1	2
C. Purchase of medicines from sidewalk vendors	1	2
D. Purchase of medicines from pharmacy	1	2
E. Consultation at dispensary	1	2
F. Consultation at health centre	1	2
G. Consultation at private practice	1	2
H. Consultation at clinic	1	2
I. Consultation at a faith-based hospital	1	2
J. Hospitalization at health centre	1	2
K. Hospitalization at public hospital	1	2
L. Hospitalization at clinic	1	2
M. Other (specify - Required) ________________________		

FILTER:
**ASK ONLY IF Q55A=1**

**Q56A. If no treatment was sought, what were the reasons?** *[Read out all options]*	Multiple answers (select any that applies)	
	Yes	No
A. Health problem was not serious enough to seek treatment	1	2
B. Could not afford cost of treatment	1	2
C. Waiting time too long or not available at time required	1	2
D. Distance to health centre/hospital/clinic too long	1	2
E. Did not expect adequate quality of treatment	1	2
F. Did not trust service/ provider	1	2
G. Discrimination/not culturally appropriate/language problems	1	2
H. Did not have time / too busy to seek treatment (work, personal or family responsibilities)	1	2
I. Service not available in area	1	2
J. Other, specify: |______________________________| [DNR]	1	2
K. Not applicable [DNR]	8	8
L. Don't know / No answer [DNR]	9	9

FILTER:
**ASK ONLY IF Q55A =1 AND (Q56AA=1 OR Q56AB=1 OR Q56AC=1, …. AD-AL=1)**

**Q56B. What is the main reason of no treatment sought?** *[Single answer] [Only ask for the main reason among the given reasons from the previous question]*	
A. Health problem was not serious enough to seek treatment	1
B. Could not afford cost of treatment	2
C. Waiting time too long or not available at time required	3
D. Distance to health centre/hospital/clinic too long	4
E. Did not expect adequate quality of treatment	5
F. Did not trust service/ provider	6
G. Discrimination/not culturally appropriate/language problems	7
H. Did not have time / too busy to seek treatment (work, personal or family responsibilities)	8
I. Service not available in area	9
J. Other, specify: |______________________________| [DNR]	10
K. Not applicable [DNR]	97
L. Don't know / No answer [DNR]	99

FILTER:
**ASK ONLY IF Q55.B=1 OR Q55.C=1 OR Q55.D=1 OR Q55.E=1 OR Q55.F=1 OR Q55.G=1 OR Q55.H=1 OR Q55.I=1 OR Q55.J=1 OR Q55.K=1 OR Q55.L=1**

**Q57. How did you or your family find the money to pay for this treatment?** [Read out] [Multiple responses]		
	Yes	No
A. No cost was charged / or paid by another source	1	2
B. Money set aside at home/ Withdrew savings	1	2
C. Sale of possessions (cattle, jewellery, tools, means of transportation…)	1	2
D. Village bank	1	2
E. Loan from a savings and credit fund	1	2
F. Loan from friends/neighbours/relatives	1	2
G. Loan from a merchant	1	2
G2. My employer paid for the treatment	1	2
H. Took a loan from employer	1	2
I. Collection taken up from co-workers	1	2
J. Received government aid	1	2
K Took a government loan	1	2
L. Other means, specify: ____________________ **[DNR]**	1	2

Block 3c: COVID-19 Questions

Basic Information about the block:

This block is asked the most knowledgeable person in the household= household head. The household head should answer about all member of the household.

**COVID_Q0. If a vaccine for COVID-19 gets introduced, would you like to get it?** *[Read out options]*	
No	1
Yes, but only for free	2
Yes, even if I have to pay for it	3
Refuse [Do not read]	8

**COVID_Q1. How much do you think you or anyone in your household is at-risk of contracting/getting COVID-19?** *[Read out options]*	
No risk at all	1
Small risk	2
Medium risk	3
High risk	4
Refuse [Do not read]	8
Don't know/ Never heard about Corona or COVID-19 [Do not read]	9

FILTER:
ASK ONLY IF COVID_Q1=1 OR COVID_Q1=2 Otherwise continue with COVID_Q3

**COVID_Q2. Why do you think that your household is not exposed to the risk of contracting covid-19?** *[Do not read options] [Multiple answers are possible]*	
Follow guidance by state authorities on preventive/protective measures (handwashing, face masks)	1
Follow guidance by traditional and religious leaders	2
Covid-19 does not exist where we live	3
Covid-19 does not exist at all	4
We are strong and healthy	5
We are young	6
The heat protects us	7

Because our ethnic group is not affected	8
We stay at home	10
COVID-19 is curable/treatable	11
Because we prevent or heal it with African medicine	12
Other (specify)	13

**COVID_Q3. In the context of your job, do you feel exposed to a risk of contracting/getting COVID-19 ?** *[Read out options]*	
No	1
Yes, very strongly	2
Yes, strongly	3
Yes, modeately	4
Yes, weakly	5

**COVID_Q4.1 What are or have been your <u>health</u> concerns or fears during the COVID-19 pandemic***? [Read options] [Multiple answers possible]*	
None [Do not read]	0
Can infect other people	1
Difficult to keep social distance	2
Limited access to health care services/no treatment	3
I cannot afford medical treatment in case of an infection with the virus	4
Epidemics related to other infectious diseases (such as measles, yellow fever, cholera, etc.)	5
Other (specify) [Do not read]	6
**COVID_Q4.2 What are or have been your <u>economic and social</u> concerns or fears during the COVID-19 pandemic?** *[Read options] [Multiple responses possible]*	
None [Do not read]	0
Rising food prices	1
Loss of customers and/or markets due to containment	2
Loss of employment/closure of business	3
Children cannot go to school or kindergarten	4
Cancellation of religious services / funerals / festivities	5
Other (specify) [Do not read]	6
**COVID_Q4.3 What are or have been your <u>political</u> concerns or fears during the COVID-19 pandemic?** *[Read options] [Multiple responses possible]*	
None [Do not read]	0
State authorities cannot manage the outbreak	1
Social cohesion is weakening in our community	2
Willingness to cooperate with others is diminishing	3
The government is becoming too powerful	4
I cannot visit my friends and family	5
Other (specify) [Do not read]	6

**COVID_Q5. What measures have you/ your household taken in response to the outbreak of the COVID-19 virus?** *[Read out options] [Multiple answers are possible]*	
Followed guidance by state authorities on preventive/protective measures (handwashing, face masks, social distancing, stay at home, self-quarantine)	1
Took my temperature	3
Took COVID-19 test at health facility	4
Stockpiled food/water	5
Participated in protest against government	6
Took African medicine	7
Other (specify)	8

**COVID_Q6. Do you think the response of your country's government to the COVID-19 virus outbreak is/was extreme, appropriate or insufficient?** *[Probe responses] [Do not read options]*	
Much too extreme	1
Somewhat extreme	2
Appropriate	3
Somewhat insufficient	4
Much too insufficient	5
Don't know [Do not read]	9

**COVID_Q7a How often have you or any member of your household done voluntary work for your community in the past 12 months such as help out others with food or cash or repairing a road?** *[Interviewer Note: Local community always refers to …] [Read out options]*	
Very frequently	1
Frequently	2
Occasionally	3
Rarely	4
Very rarely	5
Never	0
Refuse [Do not read]	8
**COVID_Q7b Since the outbreak of Covid-19, did you or any member of your household participate in voluntary work for your community…** *[Read out options]*	
…more than usual	1
…less than usual	2
…never	3
Refuse [Do not read]	8
**COVID_Q7c Since the outbreak of Covid-19, did you or any member of your household assist people in your community who were affected by the pandemic?** *[Do not read options]*	
Yes	1
No	2
Refuse [Do not read]	8
**Filter: Ask only if COVID_Q7c==1 OTHERWISE CONTINUE with COVID_Q8**	
**COVID_Q7d What kind of help did you offer?** *[Read out options] [Multiple answers are possible]*	
Cash	1
Food	2
Business support	3
Providing care for children or sick relatives	4
Emotional support and information	5
Other (specify)	6

**COVID_Q8. Since the outbreak of Covid-19 have you or any member of your household mainly received any food, cash, subsidy or other support from the government or anyone else?** *[Read out options]*	
No	1
Yes, from central government	2
Yes, from local government	3
Yes, from traditional leaders or from my church	4
Yes, from my relatives	5
Yes, from neighbours or members of my community	6
Yes, from NGOs, donors, international agencies or development projects	7
Refuse [Do not read]	8
Don't know [Do not read]	9

FILTER:
ASK ONLY IF **COVID_Q8=2 or 3 Otherwise continue with COVID_Q8b**

**COVID_Q8a. Which type of support did you receive?** *[Read out options] [Multiple answers are possible]*	
Food aid	1
Cash transfer or unemployment benefit	2
Protective equipment (Face mask, sanitation kit)	3
Business loans	4
Wage subsidy	5
Free health insurance/Subsidy of health insurance premiums	6
Subsidy of electricity/water costs	7
Reduction, deferral or suspension of the payment of certain taxes or fees	8
Other (specify)	9

FILTER:
ASK ONLY IF **COVID_Q8= 4 or 5 or 6 or 7 Otherwise continue with COVID_Q9**

**COVID_Q8b. Which type of support did you receive?** *[Read out options] [Multiple answers are possible]*	
Cash	1
Food	2
Business support	3
Watching children or sick relative	4
Emotional support and information	5
Other (specify)	6

**COVID_Q9. What main <u>problems</u> have you and your household faced since the COVID-19 virus outbreak?** *[Read out options] [Multiple answers are possible]*	
Lack of food/High food prices	1
Lack of access to medication/medical treatment	2
Decrease in household income	3
Loss of employment	4
Closure of business	5
Loss of electricity or water	6
Increase in domestic violence	7
Children cannot go to school	8

**COVID_Q10. In the recent month, has your household income been higher, the same or lower than in a typical month before the COVID-19 outbreak?** *[Read out options]*	
Higher	1
Same	2
Lower	3
Don't know [Do not read]	9

FILTER:
**ASK ONLY IF COVID_Q10=1 OR COVID_Q10=3 Otherwise continue with COVID_Q11**

	**Amount**	Don't know	Not willing to respond
**COVID_Q10a. Please provide an estimate of the amount that your household has earned more or less?**	____________ ______	9	8

**COVID_Q11. Do you think the COVID-19 pandemic will have an adverse economic impact on this country in the future?** *[Read out options]*	
Not at all	1
Somewhat in the short term, but it will return to normal soon	2
Somewhat, the economic situation will deteriorate in the coming months	3
A lot, I consider this crisis is the starting point of a major economic crisis that will exacerbate during the next months and even years	4
Don't know [Do not read]	9

FILTER:
**ASK ONLY IF COVID_Q11= 2 or 3 or 4 Otherwise continue with COVID_Q13**

**COVID_Q12. With regard to possible negative economic impact, who do you think will be affected most?** *[Read out options]*	
Everyone in this country will be equally affected by the economic impact of Covid- 19	1
The wealthy will suffer more from the economic impact of Covid-19	2
The poor will suffer more from the economic impact of Covid-19	3
Don't know [Do not read]	9

**COVID_Q13. Do you think that COVID-19 will lead to political and social tensions in this country in the future?** *[Read out options]*	
Not at all	1
Only somewhat – and only for a short while	2
Only somewhat – but for a long-time	3
A lot, - and the tensions may even worsen during the next months	4
Don't know [Do not read]	9

Random selection of the respondent

The system will pick a randomly selected person among the members of the household that have IE=1 (WORK INFORMALLY)

PLEASE ASK FOR THE RANDOMLY SELECTED HOUSEHOLD MEMBER that is IE=1

Thank you note

Thanks so much for sharing all the information with us. This is the last question for this block. For the next block we would like to talk to ______ who was selected randomly by our system . Is he/she available?

IF SELECTED PERSON IS NOT AVAILABLE MAKE APPOINTMENT AND VISIT THE HOUSEHOLD AGAIN WHEN THE PERSON IS AVAILABLE

IF PERSON IS NOT AVAILABLE AT THE 2ND VISIT SELECT RANDOMLY A NEW PERSON WITH IE=1 AND CONDUCT THE INTERVIEW WITH THIS PERSON

IF THIS PERSON IS NOT AVAILABLE STOP THE INTERVIEW

Block 4a: Basic information about selected household member

This block is asked the RANDOMLY SELECTED HOUSEHOLD MEMBER who works in the informal sector. IE=1

Introduction / Asking for consent

Good day. My name is ____________. I am from ________ an independent research organization. We are conducting a study in cooperation with Friedrich Ebert Stiftung, the International Labour Organisation and the German Development Institute. I do not represent the government or any political party. We are studying the situation of citizens in Ethiopia operating in the informal economy. Every person of the informal economy has an equal chance of being included in this study. All information will be kept confidential. Your household has been chosen by chance.

Within your household you were also selected by chance. Your answers will be confidential. They will be put together with 3336 other people we are talking to, to get an overall picture. It will be impossible to pick you out from what you say, so please feel free to tell us what you think. This interview will take about 50 minutes. There is no penalty for refusing to participate. Do you wish to proceed?

[ONLY CONTINUE IF ACCEPTED]

First, we would like to know a bit about you?

**Q65. Marital Status of Respondent. What is your marital status?**	
Married, Monogamous	1
Married, Polygamous	2
Single/Never Married	3
Single / Divorced	4
Widowed	5
Separated	6

**Q66. Religion. What is your religion, if any?**	
None	0
Christian	1
Muslim	2
Traditional / ethnic religion	3
Other (specify)	4
Refused *[Do not read]*	-998
Don’t know *[Do not read]*	-999

Q67. What is your ethnic community, cultural group, or tribe? [*Do NOT read options. Code from response] [If respondent does not identify any group on this question – that is, IF they REFUSE to answer (9998), DON'T KNOW (9999) or mention "[Ethiopian] only (9990)"– then circle 7 = Not applicable for Q85A and Q85B and continue to Q86.]*

			[insert country-specific codes]
		[Ethiopian] only, or "doesn't think of self in those terms"	9990
		Refused to answer	9998
		Don't know	9999
		Other *[Specify]:* ______________________	Post code

**Q68. What is your highest level of education?**<br><br>*[Code from answer. Do not read options]*	
No formal schooling	0
Informal schooling only (including Koranic schooling)	1
Some primary schooling	2
Primary school completed	3
Junior secondary school or some secondary school / high school	4
Secondary school / high school completed	5
Post-secondary qualifications other than university, e.g. a diploma or degree from a polytechnic or college	6
Some university	7
University completed	8
Post-graduate	9
Don't know [Do Not Read]	99

**Q69. Where were you born?** *[Read out options]*	**Yes**	**No**
A. I was born in this location	1	2
B. I was born in this district / county	1	2
C. I was born in this country (Ethiopia)	1	2
D. I was born in another country	1	2
E. Don't Know/Can't remember [Do Not Read]	9	

**FILTER:**
**ASK ONLY IF Q69B=1 OR Q69C=1 OR Q69D=1. OTHERWISE COUNTINUE TO Q72**

**Q70. What was the main reason you moved in this location?**	
….my job was transferred to here	1
… business opportunity	2
… looking for paid work	3
… family reasons	4
… attending school / training	5
… other (specify)	6
Don't know/Do not remember [Do Not Read]	9
**FILTER: SEE FILTER ABOVE**	
**THIS QUESTION SHOULD BE ASKED ONLY IF Q69B=1 OR Q69C=1 OR Q69D=1**	

**Q71. How many years ago did you move to this location? [If respondent do not know please type 9]**	
Less than 5 years ago	1
Between 5 to 10 years ago	2
Between 10 to 20 years ago	3
More than 20 years ago	4
Don't know/Do not remember [Do Not Read]	9

**Q72. Do you have plans to move within the next 5 years to another region or country?**	
No	1
Yes, I plan to move to another region inside [Ethiopia]	2
Yes, I plan to move to another country	3
Don't know/Do not remember [Do Not Read]	9

Block 4b: Informal employment – Main job of selected household member

Basic Information about the block:

This block is asked the randomly selected person in the household that works in the informal sector (that is IE=1)

Introduction:

We are now going to ask you about your employment situation. Those of you who currently do work please inform us about your current work situation. Those of you who currently do not work please inform us about your last work situation.

**Q73. Are you employed in…**	
The government or a state-owned enterprise or in the army	1
A farm	2
A private business (non-farm)	3
An international organisation (or a foreign embassy)	4
An NGO / Non Profit institution / religious organization (church etc.)	5
A household (including Domestic worker)	6
Other (specify): ________________________________	7

**Q74. In your main job you work…?**	
In (your/his/her) own business activity	1
As an employee for someone else	2
As an apprentice, intern	3
In a business operated by a household or family member	4

FILTER:
ASK ONLY IF Q74=1

**Q74.1. Do you have regular employees?**	
Yes	1
No	2

FILTER:
ASK ONLY IF Q74.1.=1

**Q74.2. How many regular employees do you employ?**					
1-2	3-5	5-9	10-20	More than 20	

**FILTER:**
**ASK ONLY IF Q74=1 AND Q73=2,3 AND Q73=7 OTHERWISE GO TO Q76**

**Q75. Is the business where you work registered with PACRA (Patents and Companies Registration Agency r):**	
Yes	1
No	2
Don't know [Do not read]	9

**Q76. Which sector does your work belong to?** *[Do not read options]*	
Agriculture, forestry and fishing	1
Mining and quarrying	2
Manufacturing	3
Electricity, gas, steam and air conditioning supply	4
Water supply; sewerage, waste management and remediation	5
Construction	6
Wholesale and retail trade; repair of motor vehicles and motorcycles	7
Transportation and storage	8
Accommodation and food service activities	9
Information and communication	10
Financial and insurance activities	11
Real estate activities	12
Professional, scientific and technical activities	13
Administrative and support service activities	14
Public administration and defence; compulsory social security	15
Education	16
Human health and social work activities	17
Arts, entertainment and recreation	18
Other service activities	19
Activities of households as employers; undifferentiated goods- and services- producing activities of households for own use	20
Activities of extraterritorial organizations and bodies	21

**Q77. How many persons including yourself are working at your place of work / in your business?** [Do not read options - ask for the number and fill in]	
1 (you alone)	1
2-4	2
5-9	3
10-19	4
20-49	5
50+	6

FILTER:
ASK ONLY IF Q74=2 OR Q74=3

**Q78. Do you have a written contract or oral agreement with your employer?**	
Yes, written contract	1
Yes, oral agreement	2
No, no agreement	3

FILTER:
ASK ONLY IF Q78=1 OR Q78=2

**Q79. Is your contract / agreement …**	
Of limited time duration	1
Permanent or without limit of time	2
For the completion of a task or tasks	3
Don't know	9

**Q80. How many hours do you usually work per week in your main job?**	
NUMBER OF HOURS	
DON'T KNOW *[Do not read]*	9999

**Q81. If you compare your average monthly income over the last six months to a value of 1,500 Birr , how do you rate your monthly income:**	
My monthly income was always below	1
My monthly income was mostly below	2
On average, my monthly income was about the same	3
My monthly income was mostly higher	4
My monthly income was always higher	5

Q82. Can you give me an estimate of your average monthly income (Birr)?
[NOTE: after paying tax and after any contributions were deducted] [Read out options]

		Range	
		From	To
	1	0	0
	2	>0	750
	3	>750	1500
	4	>1500	3000
	5	>3000	12,000
	6	>12,000	
	9	Don't	know
	8	Not willing	respond

Block 5: Citizen-state relationship of selected household member

Basic Information about the block:

For this block you stay with the person of the last one (I.e the the ramdomly selected person in the household that works in the informal sector

Introduction:

Now we would like to learn about your opinion related to the social and political life in Ethiopia

**Q83. Please tell me whether you agree or disagree with the following statement: It makes me proud to be called an Ethiopian.** *[Read out options] [Interviewer: Probe for strength of opinion.]*	
Strongly agree	1
Agree	2
Undecided [Do not read]	3
Disagree	4
Strongly Disagree	5
Don't know [Do not read]	9
Does not apply [Do not read]	7

**Q84. If the government wants to improve services to the people, what are in your opinion the sectors that the government should focus on? [Read out options]**		
	**Yes**	**No**
A. Schools and education	1	2
B. Roads and bridges	1	2
C. Police services	1	2
D. Health services	1	2
E. Electricity supply	1	2
F. Water supply	1	2
G. Pensions for the elderly	1	2
H. Food programs in times of crisis	1	2

FILTER:
ASK ONLY IF Q84A=1 OR Q84B=1 OR Q84C=1 OR Q84D=1 …OR ... Q94H=1)

**Q85. Please rank the sectors you have highlighted above?** **[***GIVE as your 1, 2 and 3 priories]*			
	First priority	Second priority	Third priortity
Answer from above	1	2	3
Answer from above	1	2	3
Answer from above	1	2	3

**Q86. How much do you think that the following organisations/institutions care about your top priorities you highlighted?** *[Read out options]*					
	Not at all	Just a little	Somewhat	A lot	Don't know/ Haven't heard *[DNR]*
A. The president	0	1	2	3	9
B. National Government	0	1	2	3	9
C. National parliament	0	1	2	3	9
D. Your local level government	0	1	2	3	9
E. Political parties	0	1	2	3	9
F. Traditional leaders	0	1	2	3	9
G. Religious leaders	0	1	2	3	9
H. Trade unions	0	1	2	3	9
I Civil society organisations	0	1	2	3	9
J. International donors	0	1	2	3	9

**Q87. Assuming the following actors are willing to work towards tackling your top priorities you highlighted, do you think that they can make a significant difference and change the situation to better through their actions?** *[Read out options]*					
	Not at all	Just a little	Somewhat	A lot	Don't know/ Haven't heard *[DNR]*
A. The president	0	1	2	3	9
B. National Government	0	1	2	3	9
C. National parliament	0	1	2	3	9
D. Your local level government	0	1	2	3	9
E. Political parties	0	1	2	3	9
F. Traditional leaders	0	1	2	3	9
G. Religious leaders	0	1	2	3	9
H. Trade unions	0	1	2	3	9
I Civil society organisations	0	1	2	3	9
J. International donors	0	1	2	3	9

**Q88. How much do you trust each of the following actors?**<br>*[Read out options]*					
	Not at all	Just a little	Somewhat	A lot	Don't know/ Haven't heard *[DNR]*
A. The president	0	1	2	3	9
B. National Government	0	1	2	3	9
C. National parliament	0	1	2	3	9
D. Your local level government	0	1	2	3	9
E. Political parties	0	1	2	3	9
F. Traditional leaders	0	1	2	3	9
G. Religious leaders	0	1	2	3	9
H. Trade unions	0	1	2	3	9
I Civil society organisations	0	1	2	3	9
J. The courts	0	1	2	3	9
K. The police	0	1	2	3	9
L. The military	0	1	2	3	9
M. International donors	0	1	2	3	9

**Q89. How willing would you be to demonstrate and protest about the followings?**<br>*[Read out options]*					
	Not at all	Just a little	Somewhat	A lot	Don't know/ Haven't heard *[DNR]*
A. Higher wages	0	1	2	3	9
B. better working conditions.	0	1	2	3	9
C. Improvement in health care.	0	1	2	3	9
D. Improvement in education	0	1	2	3	9
E. To defend democratic rights	0	1	2	3	9

**Q90. If you are faced with an important problem, which of the following persons would you approach to look for advice and support? Please name up to three**<br>*[Read out options]*	
A. A [local government councillor]	1
B. A member of [Parliament]	2
C. An official of a government agency	3
D A political party official	4
E Traditional leaders	5
F Religious leaders	6
G. Your family	8
H. Friends/ members of the community, village	9
I. Trade Unions	10

Thank you for your responses so far. Now let's talk for a moment about the kind of society and political system you would like to have in Ethiopia.

**Q91. Do you agree with the following statement?** [Read out statement]:	
Statement: The government should provide services only to those persons and households, that pay taxes and fees to the government.	
Strongly agree	1
Agree	2
Undecided [DNR]	3
Disagree	4
Strongly disagree	5
Don't know [Do not read]	9

**Q92. Do you agree with the following statement?** [Read out statement]:	
Statement: The Government should focus on services to the poor even if they are not able to pay for them.	
Strongly agree	1
Agree	2
Undecided [DNR]	3
Disagree	4
Strongly disagree	5
Don't know [Do not read]	9

**Q93. Do you agree with the following statement?** [Read out statement]:	
Statement: Only rich citizens should have to pay taxes or fees to the Government.	
Strongly agree	1
Agree	2
Undecided [DNR]	3
Disagree	4
Strongly disagree	5
Don't know [Do not read]	9

**Q94. Do you agree with the following statement?** [Read out statement]:	
Statement: Everyone who earns an income, regardless of how much it is, should have to pay some taxes or fees to the Government.	
Strongly agree	1
Agree	2
Undecided [DNR]	3
Disagree	4
Strongly disagree	5
Don't know [Do not read]	9

**Q95. Do you agree with the following statement?** [Read out statement]:	
Statement: Government should ensure that some of the wealth of the rich is used to support the poor.	
Strongly agree	1
Agree	2

Undecided [DNR]	3
Disagree	4
Strongly disagree	5
Don't know [Do not read]	9

**Q96 Do you agree with the following statement?**<br>[Read out statement]:	
Statement: The government should not interfere with the income of the rich and leave them alone.	
Strongly agree	1
Agree	2
Undecided [DNR]	3
Disagree	4
Strongly disagree	5
Don't know [Do not read]	9

Q97. Please tell me for each of the following things how essential it is for a democracy to be strong.
[Read out options]

	Not at all	Just a little	Some-what	A lot	Refuses to respond *[DNR]*	Don't know *[DNR]*
A. Governments tax the rich and subsidize the poor.	0	1	2	3	8	9
B. People choose their leaders in free elections.	0	1	2	3	8	9
C. People receive state aid	0	1	2	3	8	9
D. The army takes over when government is incompetent.	0	1	2	3	8	9
E. Civil rights protect people from state oppression.	0	1	2	3	8	9
F. The state makes people's incomes equal.	0	1	2	3	8	9
G. Everyone is allowed to say its opinion without fearing consequences	0	1	2	3	8	9
H. Women have the same rights as men.	0	1	2	3	8	9

Let´s talk about another topic. As you know the government gets some of its resources by taxing corporations and individuals to pay taxes and fees. Regarding this

Q98.1 Did you pay taxes and fees to your local government during the last year? (Read out)

(Read out)	No	Yes	Don't know (DNR)
A. Taxes on property for instance on the property of houses	0	1	9
B. License fees for example, for a bicycle, cart, business or market stall	0	1	9

**Q98.2 Do you pay taxes on a regular basis to the national government?**			
(Read out)	No	Yes	Don't know (DNR)
A. By paying value added tax for product and services you consume	0	1	9
B. By submitting a declaration on my income and paying the corresponding taxes	0	1	9

Q99. How easy or difficult do you think it is....?
[Read out options] [Interviewer: Probe for strength of opinion.]

		Very easy	Easy	Neither Difficult Nor Easy [DNR]	Difficult	Very difficult	Don't Know [DNR]
A.	To find out what taxes and fees you are supposed to pay to the government?	0	1	2	3	4	9
B.	To avoid paying the income or property taxes that people owe to government?	0	1	2	3	4	9

Q100. I am now going to ask you about a range of different actions that some people take. For each of the following, please tell me:
[Read out options]

	Not wrong at all	Wrong but Under-standable	Wrong and punishable	Don't Know [DNR]
A. Not paying fees for the services they receive from government	1	2	3	9
B. Not paying the taxes they owe on their income	1	2	3	9
C. Paying bribes to get access to health services	1	2	3	9

Q101. How often do you think that the actions we describe happen in general. For each of the following, please tell me:
[Read out options]

	Never	Sometimes	Often	Don't Know [DNR]
A. Not paying fees for the services they receive from government	1	2	3	9
B. Not paying the taxes they owe on their income	1	2	3	9
C. Paying bribes to get access to health services	1	2	3	9

**FILTER:**
**ASK ONLY IF Q98.1.A=1 OR Q98.1.B=1 OR Q98.2.A=1 OR Q98.2.B=1. IF Q98.1.A=0 OR 9 OR Q98.1.B=0 OR 9 OR Q98.2.A=0 OR 9 OR Q98.2.B.=0 OR 9 GO TO Q103.**

**Q102. Particularly in your case and that of your family. Do you consider that you get a fair amount of public services considering the money that you contribute to the state in terms of taxes, fees and other payments?**<br><br>*[Read out options] [Interviewer: Probe for strength of opinion.]- The idea is to capture whether respondents consider that a particular group is benefiting more.*	
Strongly Agree	1
Agree	2
Undecided [DNR]	3
Disagree	4
Strongly Disagree	5
Don't know/ Haven't heard [DNR]	9

Q103. What about your region, your ethnic groups and citizens of the country in general? Do you agree or disagree that they get a fair amount of public services considering the money that they contribute to the state in terms of taxes, fees and other payments?

[Read out options] *[Interviewer: Probe for strength of opinion.]* The idea is to capture whether respondents consider that a particular group is benefiting more.

	Strongly Agree	Agree	Undecided [DNR]	Disagree	Strongly Disagree	Don't know [DNR]
A. your region/county	0	1	2	3	4	9
B. your ethnic groups	0	1	2	3	4	9
C. citizens of Ethiopia in general	0	1	2	3	4	9

Three final questions on these issues

Q104. Do you think that particularly wealthy regions and wealthy individuals contribute their fair share of taxes, fees and other payments to the development of the country?

[Interviewer: Probe for strength of opinion.]

	Strongly Agree, they already pay more than enough	Agree, they pay already enough	Undecided *[DNR]*	Disagree, they should pay more	Strongly Disagree they should pay significantly more	Don't know/ *[DNR]*
A. Wealthy regions/counties	0	1	2	3	4	9
B. Wealthy individuals	0	1	2	3	4	9

**Q105. If the government decided to make you pay taxes or user fees in order to increase spending on public health care benefitting you, would you support this decision or oppose it?**<br><br>*[Interviewer: Probe for strength of opinion.]*	
Strongly oppose	1
Somewhat oppose	2
Neither support nor oppose *[Do not read]*	3
Somewhat support	4
Strongly support	5
It depends (e.g., on size of the increase) *[Do not read]*	6
Don't know *[Do not read]*	9

**Q106. Would you pay taxes or fees if the government would use this money to provide free health services for persons poorer than you?**<br><br>*[Interviewer: Probe for strength of opinion.]*	
Strongly oppose	1
Somewhat oppose	2
Neither support nor oppose *[Do not read]*	3
Somewhat support	4
Strongly support	5
It depends (e.g., on size of the increase) *[Do not read]*	6
Don't know *[Do not read]*	9

Block 6: Use of health services by selected household member

Basic Information about the block:

For this block you stay with the person of the last one (I.e the randomly selected person in the household that works in the informal sector

Introduction:

Now we would like to learn about your opinion related to the use of health services

**I would like to ask you about your experience the last time you sought medical treatment at a health facility…**					Read out: Single mention only	
	Very good	Good	Poor	Very poor	DK	No contact
**Q107. Please rate the quality of patient reception**	1	2	3	4	9	7
**Q108. Please rate the competency of the medical staff**	1	2	3	4	9	7
**Q109. How easy or difficult was it to obtain the medical care you needed? [Read out options]**	Very easy	Easy	Difficult	Very difficult	DK	No contact
	1	2	3	4	9	7
**Q110. How long did it take you to receive the medical care that you needed? Was it right away, after a short time, after a long time, or never?**	Right away	After a short time	After a long time	Never	DK	No contact
	1	2	3	4	9	7
**Q111. Did you have to pay a bribe, give a gift, or do a favour for a health worker or clinic or hospital staff in order to get the medical care you needed?**	Yes		No		DK	No contact
	1		2		9	7

**Q112. If you compare public health with private health services, which one is better with regard to:** *[Read out options]*					
	Public health services	Private health services	Same *[Do not read]*	None *[Do not read]*	Don't know *[Do not read]*
A. Quality of patient reception	1	2	3	4	9
B. Competency of the medical staff	1	2	3	4	9
C. Availability of equipment	1	2	3	4	9
D. Availability of medicine	1	2	3	4	9
E. Accessibility of health facilities (time & cost to get there)	1	2	3	4	9

Q113. Looking at the cost of health services in public and private health facilities, which would you say provides more benefits for the same amount of money paid?	
In public health centers	1
in private health centers	2
Similarly in both, there aren't big differences *[Do not read]*	3
None of them *[Do not read]*	4
Don't know *[Do not read]*	9

Q114. Are you covered by any health insurance? *[If there are more than one health insurance scheme, please ask for the main one. Main health insurance is the one that have been used mostly.]*	
Yes, public health scheme such as CBHI (Community-based health insurance scheme)	1
Yes, private health insurance	2
Yes, micro-insurance scheme	3
Previously had health insurance but dropped out	4
Never had health insurance	5

FILTER:
THIS QUESTION SHOULD ONLY BE ASKED IF Q74=2 or 3 AND Q114=1 OR 2 OR 3

Q114.1. Are you the main contributor to the scheme or are you covered indirectly through another family member	
I am the main contributor	1
Another household member (including the household head) is the main contributor	2
My/his/her employer is the main contributor	3
Don't know *[Do not read]*	9

FILTER:
ASK ONLY IF Q74=2 or 3 AND Q114.1=1 OR Q114=4 OR 5

Q114.2. Does your employer supplement/add to your salary to cover the cost of the health insurance	
YES	1
No	2
I do not know	9

FILTER:
ASK ONLY IF Q74=2 or 3 AND Q114=4 OR 5

Q114.3. Does your employer pay for the medical treatment if you have a work-related accident, injury or sickness?	
YES	1
No	2
I do not know	9

FILTER:
**ASK ONLY IF Q74=2 or 3 AND Q114=4 OR 5**

**Q114.4. Does your employer pay for the medical treatment if you have NO work-related accident, injury or sickness?**	
YES	1
No	2
I do not know	9

FILTER:
**ASK ONLY IF Q114=4**

**Q114.5. What were the reasons for you to leave the health insurance?**		
	YES	NO
I left my job and my new employer did not pay the premium	1	2
I left my job and found no new employer	1	2
I continued in my job but the employer stopped paying the premium	1	2
I could not afford to pay the premium any more	1	2
I was not satisfied with the services of the health insurance	1	2
Other reason: pls explain ____		

FILTER:
**ASK ONLY IF Q114=4 OR Q114=5 OTHERWISE MOVE TO Q117**

**Q115. Would you be interested in joining a health insurance scheme? [Explain that this means contributing to a scheme on a regular basis in order to receive a financial compensation when needed]**		
Yes	1	**Go to Q118**
No	2	
Refused *[Do not read]*	8	
Don't know *[Do not read]*	9	

FILTER:
**Filter: If Q115=2**

**Q116. For which main reason are you not interested to join a health insurance scheme?** *[Read out options]*	
I do not have enough financial resources	1
I do not trust in health insurance scheme	2
I do not know anything about the way they work or proceed	3
Other (specify)______________________	4
Non applicable *[Do not read]*	7
Do not know *[Do not Read]*	9

FILTER:
ASK ONLY IF Q114=1 OR Q114=2 OR Q114=3

**Q117. Overall, how satisfied are you with the services provided by the health insurance scheme you are affilited with?** *[Read out options- If asked, tell interviewee we received the informaiton from the household head]*	
Very satisfied	4
Fairly satisfied	3
Not very satisfied	2
Not at all satisfied	1
Non applicable *[Do not read]*	7
Do not know *[Do not Read]*	9

FILTER:
ASK ONLY Q115=1 OTHERWISE GO TO NEXT BLOCK

**Q118A. With what frequency would you prefer to pay health insurance premiums?**		
		**Q118B.** *[Interviewer: if Q118A is 1 or 2, ask]* **What month would you prefer to pay premiums?**
Once per year	1	____________________
Once every six months	2	____________________
Once every three months	3	
Once a month	4	
Once a week	5	
Daily	6	
Not applicable	7	

FILTER:
THIS QUESTION SHOULD ONLY BE ASKED IF Q115=1 (SEE FILTER ABOVE)

**Q119. What premiums amount would you be prepared to pay with the frequency you have indicated above?**	Codes
Amount per person [Unit to be indicated]	|_|_|_|_|
None	1
Don't know *[Do not read]*	7
No answer *[Do not read]*	9

Block 7: Organizations and Trade Unions

Block 7a: Participation in CSOs

Basic Information about the block:

For this block you stay with the person of the last one (i.e the randomly selected person in the household that works in the informal sector

Use the following definition/examples of groups:

- **Cooperative:** an autonomous association of persons united voluntarily to meet their common economic, social, and cultural needs and aspirations through a jointly owned and democratically-controlled enterprise. (ICA definition) (Cooperatives are usually registered);
- **Credit union:** as above but providing financial services only (savings and credit);
- **Tontine/Chama:** a group of individuals who agree to meet for a defined period in order to save and borrow together. Tontines are usually not registered. Credit unions maintain individual accounts for each member whereas tontines disburse the sums contributed by all members to one of them, in one amount and on a rotating basis.
- **Trade union**: an organized association of workers in a trade, group of trades, or profession, formed to protect and further their rights and interests. Trade union must be registered and must have a structure according to statutes
- **Mutual health group/community-based health insurance**: democratic institutions founded on the principles of mutual assistance and solidarity. Members pay a monthly or yearly fee and are entitled to the partial or full reimbursement of health-related expenditures. Mutuals are usually registered.
- **Political party**: an organised group of people with at least roughly similar political aims and opinions, that seeks to influence public policy by getting its candidates elected to public office.
- **Social movement**: a network of informal interactions between several individuals or groups engaged in a political or cultural conflict, on the basis of a shared collective identity
- **Professional organization**: usually a non-profit organization seeking to further a particular profession, the interests of individuals engaged in that profession and the public interest.
- **Cultural group:** a group of individuals who collectively undertake cultural activities (music, arts, singing, dancing etc.)
- **Religious group**: A set of individuals whose identity as such is distinctive in terms of common religious creed, beliefs, doctrines, practices, or rituals
- **Sports association**: a group of individuals who collectively do sports, such as football, basketball, etc.
- **Association:** any other group of people organized for a joint purpose. The purpose of the association can be derived from its functions (see question 3). However, many associations are not registered and remain informal. Begin with the more specific types of groups and tick "association" only if none of them applies.

Q120. Do you belong to one of the following groups?
[Read out options] [Multiple responses]

**I will read out the names of four groups**	YES	NO
**a.** Cooperative	1	2
**b.** Credit union	1	2

**Q120. Do you belong to one of the following groups?** [Read out options] [Multiple responses]		
**I will read out the names of four groups**	YES	NO
**c.** Tontine or ROSCCA	1	2
**d.** Mutual health benefit group	1	2
**I will read out now the names of four other types of groups**		
**e.** Trade union	1	2
**f.** Political party	1	2
**g.** Religious group	1	2
**h.** Professional organization	1	2
**I have now another 4 types of groups which I read-out**		
**i.** Cultural group	1	2
**j.** Social movement	1	2
**k.** Neighbourhood or Residence Association	1	2
**l.** Any other type of association or self-help group not mentioned so far	1	2
[only read if the respond to the item previously listed is always No]. **Is it correct that you are not a member of any group**		
**m.** I am not a member of a group	1	2

**FILTER:**
**IF Q120m=1 GO TO Q120.1.**

**Q120.1. Did you belong to one of the groups we just mentioned in the past?** [Read out options] [Multiple responses]		
	YES	NO
**The 1st four groups**		
a. Cooperative	1	2
b. Credit union	1	2
c. Tontine or ROSCCA	1	2
d. Mutual health benefit group	1	2
**The 2nd four groups**		
e. Trade union	1	2
f. Political party	1	2
g. Religious group	1	2
h. Professional organization	1	2
**The 3rd. four groups**		
i. Cultural group	1	2
j. Social movement	1	2
k. Neighbourhood or Residence Association	1	2
l. Any other type of association or self-help group not mentioned so far	1	2
[only read if the respond to the item previously listed is always No]. **Is it correct that you never were a member of any group before?**		
m. I was never a member of any group	1	2

**IF Q120.1.a-l=1 ask Q120.2. // IF Q120.1.m.=1 GO TO Block 7 (Views on Trade Unions)**

**Q120.2. You previously belonged to a group and you have left it. What were your reasons for leaving the group?** [Read out options] [Multiple responses]		
	YES	NO
1. I moved to a different region	1	2
2. The group dissolved and all members left the group	1	2
3. I could not afford to pay the member fees anymore	1	2
4. I did not need the service of the group anymore	1	2
5. The group did not help me when I needed help	1	2
6. My group was inefficient, this is why I left it	1	2
7. Other reason: pls. explain ______________________________		

All Q120m=1 GO TO Block 7b (Views on Trade Unions)

**Q121. Among the groups in which you are a member: Which is the most important to you?** [Do not read out options-]	
	[___________insert name of most important group]
**a.** Cooperative	1
**b.** Credit union	2
**c.** Chilimba/village bank	3
**d.** Trade union	4
**e.** Mutual health benefit group	5
**f.** Political party	6
**g.** Social movement	7
**h.** Professional organization	8
**i.** Cultural group	9
**j.** Religious group	10
**k.** Neighbourhood or Residence Association	11
**l.** Any other type of association or self-help group not mentioned so far	12

**Q122. What is your role in this group?** *[Read out and provide examples of what is meant by the categories below. Insert answers into the table below. Multiple roles per group are possible, for example "founding"+"active"+"board"]:*					
	Founding member	Board member	Chair/Leader/ President	Active member	Passive member
[____________insert name of most important group]	1	2	3	4	5

**Q123. What are the reasons for you to join this group?**<br><br>[You could ask "Why did you join the group" or "what does the group do for you" or "I use the group to ….". Tick the relevant cells of the table below. Multiple choice possible.]<br>[The focus of this question lies on social and societal/political functions. If the respondent cites only financial and economic/business functions (shaded in blue) ask whether the group carries out *in addition* social and societal/political functions as well.]	
	[____________)<br>*insert name of most important group*
Financial services	1
Economic/business/professional services	2
Social Services	3
Societal/Political Functions	4
Others	5
Please specify	

Definitions of the categories [the interviewer could read out some if needed]:

**Financial services:**<br>Groups that provide saving money (putting my savings into an account), borrowing money (getting a loan from my group) or saving money together through a tontine.
**Economic/business/professional services:**<br>Group members working together on common land (joint agricultural production) or in common premises (joint manufacturing) or they do joint agricultural work (land preparation, harvesting etc.) or they work together for marketing of crops, livestock, products or services.
**Social Services:**<br>Groups that provide financial aid in case of an emergency (mutual assistance fund) or groups that provide community-based health centre/insurance or community-based care services (care for the elderly, children, disabled, sick persons).
**Societal/Political Functions:**<br>Groups that are negotiating with local authorities [for example: daily fees for a market stall; license rates for taxi drivers; issuing of ID cards from informal economy operators] or groups that are bargaining with employers or groups that organizes public protests or public campaigns to demand social, societal or political change.

**Q124. How did the group support you after health, work or other related shocks?**<br>[Read out options] [Multiple responses]	
	A. *[____________) insert name of most important group]*
No support at all	1
Helped me to get a loan	2
Gave me money	3
Helped me with my work (temporarily)	4
Helped me in-kind (support me with inputs for farming, housing etc)	5
Other (Specify) __________	__________
Not applicable [do not read]	-888

**Q125. Thinking about the groups you mentioned. How many persons would you say (estimate) are members of the groups?**						
	Less than 10	10 to 50	51 to 100	101 to 1,000	Above 1,000	Not applicable
[____________)_insert name of most important group]	1	2	3	4	5	7

**Q126. Looking at the composition of your groups, would you say the majority of the members are of the same ……**<br><br>[. Multiple options per group are possible]										
	Ethnic group, tribe	Faith, religion	Professional occupation	Business community	Age group, generation	Income level, class	Location (market, illage)	Country of origin (for migrants)	Same political thinking	Not applicable
[____insert name of most important group]	1	2	3	4	5	6	7	8	9	77

**Q127. Is your group officially registered or recognized?**<br><br>[Provide examples: "does your group has a registration slip? Is it recognized by the local authority {mayor, district officer, market authority, relevant ministry}]. The group…					
	Has an official registration	Is recognized by authorities, but does not have a receipt	Is not officially registered nor recognized	Not applicable	Does not know *[Do not read]*
[____________insert name of most important group]	1	2	3	7	9

**Q128. Did you or do you have to pay money to become or remain a member of the group?**<br><br>[Options include: subscribe to a share capital; pay weekly, monthly, yearly fees; have a percentage deducted from the transaction with the group; make voluntary contributions. Multiple options are possible.]								
	Paid entry fee	Bought a member share	Pay a regular fee	Pay an irregular fee	Make voluntary contributions	%deducted or added from my group dealings	Pay nothing	Not applicable
[____insert name of most important group]	1	2	3	4	5	7	8	77

**Q129. Does your group belong to a secondary body, such as a union, an association, a representational body, an alliance, a network, a federation or confederation?**<br><br>if the answer is "yes", ask for the name of the high-level structure].					
	Belongs to a higher-level structure	Does not belong to a higher-level structure	Not applicable	Does not know *[Do not read]*	Name of higher-level structure
[__________insert name of most important group]	1	2	7	9	**Q130A.**<br>[If name is not known, write Don't know]<br>____________________

**Q131. Does your group keep accounts?**<br>*[Interviewer: ask if respondent keeps a cash register, membership accounts, sales book, or any other form of accounts].*				
	Keeps accounts	Does not keep accounts	Not applicable	Does not know *[Do not read]*
[__________insert name of most important group]	1	2	7	9

**Q132. Does the group operate permanently or temporarily?**					
	Operates permanently	Operates seasonally (e.g. during harvesting time)	Operates when a need arises [ad hoc]	Not applicable	Does not know *[Do not read]*
[__________insert name of most important group]	1	2	3	7	9

**Q133. How many years does the group exist?**	
	[_________)_ insert name of most important group]
Number of years *(Interviewer: if less than 12 months, code 0 (less than a year)*	
Not applicable	7
Does not know *[Do not read]*	9

**Q134. What is the geographic coverage of the group?**<br><br>[interviewer: by "geographic coverage" we mean the area of operation and membership of the group].							
	A certain market or part of a market	A village or a quarter of a town	A district or administrativ e department	A province, county or federal state	The entire nation	Not applic able	Does not know *[Do not read]*
A. 1st Group *[___________insert name of 1st group]*	1	2	3	4	5	7	9

**Q135. How satisfied are you with the services and performance of your group?**<br><br>[Assess this aspect on a scale of 1 to 5, where "5" signifies "totally satisfication" and "1" signifies "totally dissatified"; "0" signifies "not applicable"]. *[Interviewer: Probe for strength of opinion.]*-						
	Very dissatisfied	Dissatisfie d	Neither Nor *[DNR]*	Satisfied	Very satisfied	Not applicable
[___________insert name of most important group]						
A. The services I get from my group are useful	1	2	3	4	5	7
B. The services I get from my group are timely	1	2	3	4	5	7
C. The leadership of my group is competent	1	2	3	4	5	7
D. The leadership of my group is honest	1	2	3	4	5	7
E. The membership fees I have to pay are reasonable	1	2	3	4	5	7

Block 7b: Views on TUs

**Q136. Have you ever heard about Trade Unions and what they do?**	
A. I have not heard about TUs	1
B. I have heard about TUs but do not know what they are doing	2
C. I know a little about trade unions	3
D. I have heard about trade unions and I know what they are doing	4

FILTER:
FROM THIS POINT ON ASK ONLY IF (Q120e=1) OR (Q136=B OR C OR D)<br>OTHERWISE END THE SURVEY (INTERVIEWER- DO NOT FORGET THE LAST BLOCK)

Q137. We have listed below several statements about trade unions and want to know, if you agree or disagree. Please tell us for each statement, if you fully agree; mostly agree; partly agree/partly disagree; mostly disagree; full disagree *[Interviewer: Probe for strength of opinion.]-*

	Fully disagree	Mostly disagree	Partly agree/partly disagree [DNR]	Mostly agree	Fully agree	Don't know/ No answer [DNR]
A. In General, TUs are important organizations	1	2	3	4	5	9
B. TUs in/ETHIOPIA are inefficient	1	2	3	4	5	9
C. TUs in /ETHIOPIA help to improve the social situation for many people	1	2	3	4	5	9
D. TUs in /ETHIOPIA are corrupt	1	2	3	4	5	9
E. It would be good if TUs in /ETHIOPIA become stronger	1	2	3	4	5	9
F. TUs in ETHIOPIA should be banned	1	2	3	4	5	9

Q138. Do you agree with the following statement?
[Interviewer: Probe for strength of opinion.]

	Fully disagree	Mostly disagree	Partly agree/ partly disagree [DNR]	Mostly agree	Fully agree	Don't know/ No answer [DNR]
TUs in ETHIOPIA are independent from the government?"	1	2	3	4	5	9

Q139. Do you agree that Trade Unions improve the situation of the following groups:
[Interviewer: Probe for strength of opinion.]

	Fully disagree	Mostly disagree	Partly agree/ partly disagree [DNR]	Mostly agree	Fully agree	Don't know/ No answer [DNR]
a. The Poor	1	2	3	4	5	9
b. Workers in the informal economy	1	2	3	4	5	9
c. The Farmers	1	2	3	4	5	9
d. The Unemployed	1	2	3	4	5	9
e. The employees of the Government	1	2	3	4	5	9
f. The Government	1	2	3	4	5	9
g. The employers in the private sector	1	2	3	4	5	9
h. Own account workers	1	2	3	4	5	9
i. Everybody	1	2	3	4	5	9

FILTER:
ASK ONLY IF Q136=B OR C OR D

Q140. Talking specifically about yourself,……			
	Yes	*No*	*Don't know/ No answer*
A. Have you ever been contacted by a trade unionist?	*1*	*2*	9
B. Did you ever participate in any trade union activity?"	*1*	*2*	9
C. Do you see a benefit for yourself in what TUs are doing?	*1*	*2*	9
D. If a trade union calls for a demonstration, could you consider to participate?	*1*	*2*	9
E. Did you ever consider of becoming a member of a TU?	*1*	*2*	9

FILTER:
ASK ONLY IF (Q122=1 OR 2 OR 3 OR 4 OR 5) AND (Q136=B OR C OR D). OTHERWISE GO TO NEXT BLOCK

Q141. We want to know your views about a possible cooperation between your group and trade unions:

[Interviewer: Probe for strength of opinion.]-

	Fully disagree	Mostly disagree	Partly agree/ partly disagree [DNR]	Mostly agree	Fully agree	Don't know/ No answer [DNR]
A. Do you believe that your group should ask TUs for support?"	1	2	3	4	5	9
B. Do you think that your group should become an affiliate to an umbrella trade union	1	2	3	4	5	9

FILTER:
ASK ONLY IF (Q122=1 OR 2 OR 3 OR 4) AND (Q136=B OR C OR D). OTHERWISE GO TO NEXT BLOCK

Q142 Do you disagree or agree that trade unions could provide the following services to your group(s)?

[Interviewer: Probe for strength of opinion.]-

	Fully disagree	Mostly disagree	Partly agree/ partly disagree [DNR]	Mostly agree	Fully agree	Don't know/ No answer [DNR]
A. Help us in recruiting more members for our own organization	1	2	3	4	5	9
B. Financial support for our secretariat	1	2	3	4	5	9
C. Financial support for our own activities	1	2	3	4	5	9
D. Professional training for our own members	1	2	3	4	5	9

E. Advise/counseling in legal matters including representation in the courts	1	2	3	4	5	9
F. Help us in collective bargaining with local authorities	1	2	3	4	5	9
G. Support us to organize a demonstration	1	2	3	4	5	9
H. Help us to get a health insurance scheme for our members	1	2	3	4	5	9
I. Help us that government listens to our problems and needs	1	2	3	4	5	9
J. Help us to get our own seat in the social dialogue with government	1	2	3	4	5	9
K. Help us to formalize our activity	1	2	3	4	5	9
L. Help us to improve our working conditions	1	2	3	4	5	9
M. Help us to administer our group affairs	1	2	3	4	5	9
N. After becoming a member of a TU my group should be dissolved	1	2	3	4	5	9

FILTER:
ASK ONLY IF (Q122=1 OR 2 OR 3 OR 4) AND (Q136=B OR C OR D). OTHERWISE GO TO NEXT BLOCK

Q143. If your organization would become an affiliate of a trade union, what should be the financial relation between your group and the trade union?

[Read out options]

	Yes	No	Don't know/ No answer [DNR]
A. My group should pay member fees to the trade unions and not demand a reduction	1	2	9
B. My group should be granted a discount as my organization is too poor to pay full fees to the trade unions	1	2	9
C. The trade union should not ask for fees but provide us with services for free	1	2	9

Thank you note:
Thanks so much for your valuable time and important insights. This was very helpful!

[END INTERVIEW -- DON'T FORGET TO COMPLETE NEXT BLOCK

ALL SUBSEQUENT QUESTIONS SHOULD BE ANSWERED BY THE INTERVIEWER AFTER THE INTERVIEW IS CONCLUDED]

Block 8: Closing information [to be filled by the interviewer]

Q144. What proportion of the questions do you feel the first respondent had difficulty answering?	
All	4
Most	3
Some	2
Few	1
None	0

Q145. What proportion of the questions do you feel the second respondent had difficulty answering?	
All	4
Most	3
Some	2
Few	1
None	0
Not applicable (there was only one respondent)	9

Q146. What was the first respondent's attitude toward you during the interview?	
Friendly	1
Neither friendly, nor hostile something in between	2
Hostile	3

Q147. If there was a second respondent. What was the second respondent's attitude toward you during the interview?	
Friendly	1
Neither friendly, nor hostile something in between	2
Hostile	3
Not applicable (there was only one respondent)	9

Q148. What was the primary language used in the interview?

Q149. ENDTIME	Hour		Minute	
Time interview ended *[Interviewer: Enter hour and minute, use 24 hr. cloc*				

INTERVIEWER DETAILS

**Q150. Interviewer's name** *[Write in]*					
**Q151. Interviewer's number**					
**Q152. Interviewer's age**					

**Q153. Interviewer's gender**	
Male	1
Female	2

**Q154. Do you come from a rural or urban area?**	
Rural	1
Urban	2

**Q155. Interviewer's home language:**							
				Insert			
				Country			
				Specific			
				Codes			
		Other *[Specify]:* ____________________	Post code				

**Q156. Interviewer's ethnic group / tribe:**							
				Insert			
				Country			
				Specific			
				Codes			
		Other *[Specify]:* ____________________	Post code				

**Q157. Interviewer's highest level of education**	
Primary school completed	3
Some secondary school / high school	4
Secondary school / High school completed	5
Post-secondary qualifications other than university, e.g. a diploma or degree from a polytechnic or college	6
Some university	7
University completed	8
Post-graduate	9

Q158. SIGNATURE

Q158. INTERVIEWER: Do you have any other comments on the interview? For example, did anything else significant happen during the interview?

No	0
Yes: *[Explain]* ____________________ ____________________ ____________________	1

Q159. INTERVIEWER: I hereby certify that this interview was conducted in accordance with instructions received during training. All responses recorded here are those of the respondent who was chosen by the appropriate selection method.

INTERVIEWER SIGNATURE:

____

SUPERVISOR: Do you have any other comments on the interview? For example, did anything else significant happen during the interview?

No	0
Yes: *[Explain]* ____	1

Q160. SUPERVISOR: I hereby certify that this interview was conducted in accordance with instructions given to interviewers during training. All responses have been checked for completeness and accuracy.

SUPERVISOR SIGNATURE: ____

[Supervisor Use Only]

**Household back-checked?**	
Yes	1
No	2
